# Characterization of Virus-Inducible Orchid Argonaute 5b Promoter and Its Functional Characterization in *Nicotiana benthamiana* during Virus Infection

**DOI:** 10.3390/ijms23179825

**Published:** 2022-08-29

**Authors:** Kotapati Kasi Viswanath, Song-Yi Kuo, Ying-Wen Huang, Nai-Wen Tsao, Chung-Chi Hu, Na-Sheng Lin, Sheng-Yang Wang, Yau-Heiu Hsu

**Affiliations:** 1 Graduate Institute of Biotechnology, National Chung Hsing University, Taichung 40227, Taiwan; 2 Advanced Plant Biotechnology Center, National Chung Hsing University, Taichung 40227, Taiwan; 3 Department of Forestry, National Chung Hsing University, Taichung 40227, Taiwan; 4 Institute of Plant and Microbial Biology, Academia Sinica, Taipei 11529, Taiwan

**Keywords:** Argonautes 5b, CymMV, ORSV, MeJA, NbMYB30, SA

## Abstract

Plant ARGONAUTES (AGOs) play a significant role in the defense against viral infection. Previously, we have demonstrated that AGO5s encoded in *Phalaenopsis aphrodite* subsp. *formosana* (PaAGO5s) took an indispensable part in defense against major viruses. To understand the underlying defense mechanism, we cloned PaAGO5s promoters (*p*PaAGO5s) and analyzed their activity in transgenic *Nicotiana benthamiana* using β-glucuronidase (GUS) as a reporter gene. GUS activity analyses revealed that during *Cymbidium mosaic virus* (CymMV) and *Odontoglossum ringspot virus* (ORSV) infections, *p*PaAGO5b activity was significantly increased compared to *p*PaAGO5a and *p*PaAGO5c. Analysis of *p*PaAGO5b 5′-deletion revealed that *p*PaAGO5b_941 has higher activity during virus infection. Further, yeast one-hybrid analysis showed that the transcription factor NbMYB30 physically interacted with *p*PaAGO5b_941 to enhance its activity. Overexpression and silencing of *NbMYB30* resulted in up- and downregulation of *GUS* expression, respectively. Exogenous application and endogenous measurement of phytohormones have shown that methyl jasmonate and salicylic acid respond to viral infections. NbMYB30 overexpression and its closest related protein, PaMYB30, in *P. aphrodite* subsp. *formosana* reduced CymMV accumulation in *P. aphrodite* subsp. *formosana*. Based on these discoveries, this study uncovers the interaction between virus-responsive promoter and the corresponding transcription factor in plants.

## 1. Introduction

Emerging plant viruses and their pathogenicity are major issues in plant virology [1]. RNA silencing is considered to be an important mechanism to help plants fight against viruses [2,3]. The siRNA from virus or miRNA from host is loaded into the RAN-induced silencing complex (RISC). The binding preference of argonaute proteins to small RNA determines the small RNA species that can be loaded into RISC. With the proper combination of small RNA and AGO proteins, RISC can be led to designated targets to regulate or participate in the defense system against viruses [4,5].

Accumulating evidence indicates that several plant AGOs have antiviral functions. For example, the potential antiviral activities of AtAGO1 and AtAGO7 against *Turnip crinkle virus* (TCV) mutants [6], AtAGO2 and AtAGO5 against *Potexvirus* X (PVX) [7], and AtAGO2 and AtAGO3 against *Bamboo mosaic virus* (BaMV) infection have been reported in *Arabidopsis* [8]. In rice, OsAGO1 and OsAGO18 exhibit antiviral activity against the *Rice stripe virus* (RSV) and *Rice dwarf virus* (RDV) [9]. In *Nicotiana benthamiana*, NbAGO1 and NbAGO2 show antiviral activity against *Tomato ringspot virus* (ToRSV), *Tomato bushy stunt virus* (TBSV) P19 suppressor mutants [10,11], and *Tobacco mosaic virus* (TMV) [12]. *N. benthamiana* AGO4 is involved in PVX resistance [13], and NbAGO5 is also able to bind *Cucumber mosaic virus* (CMV) vsiRNAs [14], signifying its role in antiviral defense. Our previous study identified that NbAGO1-restricted BaMV and NbAGO10 might compete with NbAGO1 for BaMV-derived small interfering RNAs (vsiRNAs) to protect BaMV from NbAGO1-mediated antiviral RNA silencing in *N. benthamiana* [15].

Plant hormones, such as salicylic acid (SA), jasmonic acid (JA), and abscisic acid (ABA), play a crucial role in regulating the defense mechanism against pathogen infection [16]. In addition, increasing evidence has shown the role of phytohormones as mediators of RNA silencing [17,18]. For example, ABA induces resistance to PVX and BaMV [8] by regulating AtAGO2 and AtAGO3 in *Arabidopsis*. NbAGO2 was transcriptionally induced by methyl salicylate treatment and TMV infection in *N. benthamiana* [12]. In *Arabidopsis*, treatment with SA and ABA together slightly increased the expression of AtAGO2, whereas ABA treatment alone increased the expression of AtAGO4, AtAGO6, and AtAGO7 [19]. The transcriptional activity of AtAGO1 and OsAGO18 has been shown to increase during ABA [20] and JA treatment [21], respectively. In addition to phytohormones, the transcriptional regulation of stress-responsive genes through binding transcription factors (TFs) is an essential part of the plant response to viral infection. Various TFs, including NAC (NAM, ATAF1,2 and CUC2) [22,23,24], MYB (myeloblastosis related) [24,25,26], AP2/ERF (APETALA2/ethylene-responsive factor) [27], WRKY [28], and bZIP (basic leucine zipper) [29,30] are involved in viral stress responses. 

Orchids have a unique status in floricultural crops that are commercially grown worldwide. The moth orchid, *P. aphrodite* subsp. *formosana* is renowned for its extraordinary floral diversity, implying complex flower color development, and is one of the most valuable research materials for molecular biology studies [31]. However, the farming and marketability of orchids have been seriously hampered by various pathogens, specifically viruses, which are not efficiently controlled by pesticide applications [32]. Two major infectious viruses of orchids, *Cymbidium mosaic virus* (CymMV) and *Odontoglossum ringspot virus* (ORSV), pose severe threats to the orchid industry. Recently, we identified that the *P. aphrodite* subsp. *formosana* AGO5 (PaAGO5) protein family plays a vital role in defense against CymMV and ORSV [33]. Specifically, PaAGO5b enhances resistance against CymMV and ORSV. More recently, we explored the induced activity of TF NbNAC42 on NbAGO5 under BaMV infection in *N. benthamiana* (provisionally accepted). To date, little is known about the effect of viral infection on the AGO gene promoters. Moreover, the extent to which TFs and phytohormones affect the expression and function of AGOs during viral infections remains to be determined.

To uncover the inducing effect of the viruses on the AGO promoter, we selected the PaAGO5b promoter for the current study because of the significant upregulation of PaAGO5b transcripts during CymMV and ORSV infection. Hence, the objective of this study was to identify the factors that activate PaAGO5b expression by exploring PaAGO5b promoter activity in transgenic *N. benthamiana*.

## 2. Results

### 2.1. Identification and In Silico Analysis of PaAGO5 Promoters

We cloned and sequenced *P. aphrodite* subsp. *formosana* AGO5a, AGO5b, and AGO5c putative promoter sequences with 2759-, 2029-, and 2589-bp lengths. TSS and the 5′-UTR were identified (Supplementary Figure S1). A BLAST search revealed that *P. aphrodite* subsp. *formosana* AGO5a, AGO5b, and AGO5c promoter sequences showed 95.23%, 99.74%, and 98.98% identity with those of *P. equestris*. The cloned promoter sequences were named *p*PaAGO5a, *p*PaAGO5b, and *p*PaAGO5c (*p*-indicates promoter) and analyzed using PlantCARE to identify possible *cis*-acting elements. We found numerous *cis*-acting elements connected to plant growth and development, phytohormone responses, and stress responses, including core transcriptional regulatory elements (i.e., TATA-box and CAAT-box) (Supplementary Tables S1–S3).

### 2.2. Activity of pPaAGO5s in P. aphrodite subsp. formosana and N. benthamiana during CymMV and ORSV Infection

**Figure 1:**
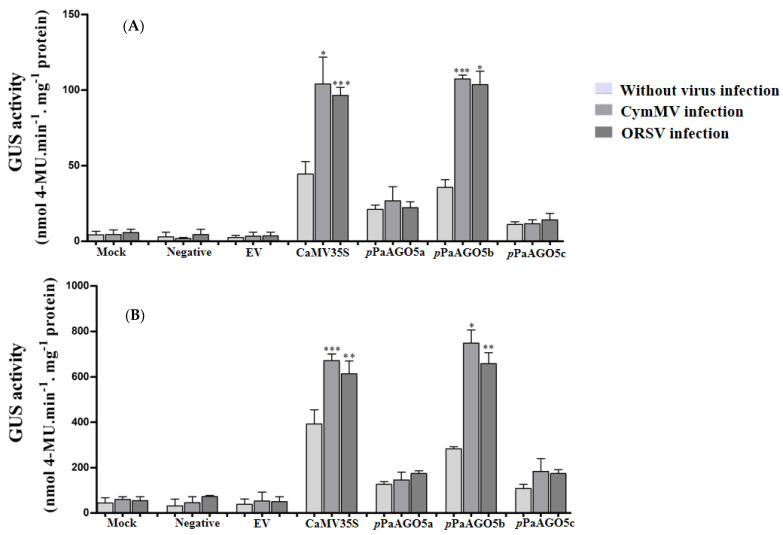
GUS fluorescent quantitative analysis of the *p*PaAGO5a, *p*PaAGO5b, and *p*PaAGO5c. The GUS activity in the wild-type (**A**) *P. aphrodite* subsp. *formosana* and (**B**) *N. benthamiana* leaves during CymMV and ORSV infection at 3 days post-inoculation (dpi). Each promoter GUS construct was assayed at least three times in four independent experiments. The GUS activity, 4-MU nmol-produced min^−1^ mg^−1^ protein, is represented as the mean ± SD of each mock (infiltration buffer only), negative and empty vector (EV; pKn), CaMV 35S, *p*PaAGO5a, *p*PaAGO5b, and *p*PaAGO5c. Data are mean ± SD, * *p* < 0.05, ** *p* < 0.01, *** *p* < 0.001 by Student’s *t*-test, respectively.

To characterize the promoter activities, GUS was used as a reporter in the following experiments. Recombinant constructs pCAMBIA-*p*PaAGO5a::GUS, pCAMBIA-*p*PaAGO5b::GUS, and pCAMBIA-*p*PaAGO5c::GUS (Supplementary Figure S2) were agroinfiltrated for transient expression in WT *P. aphrodite* subsp. *formosana* and *N. benthamiana*. At 3 dpi, leaves were detached to check for GUS activity. Histochemical staining data from *P. aphrodite* subsp. *formosana* showed visible accumulation of blue dye in *p*PaAGO5b leaf discs and low and basal activity levels in *p*PaAGO5a and *p*PaAGO5c leaf discs without CymMV or ORSV infection (Supplementary Figure S3). However, during CymMV or ORSV infection, the blue color intensity was higher in the leaf discs of *p*PaAGO5b than in *p*PaAGO5a and *p*PaAGO5c (Supplementary Figure S3). The histochemical results were further validated by fluorometric quantification of GUS activity. Among the three promoters, *p*PaAGO5b showed the highest activity in *P. aphrodite* subsp. *formosana* (Figure 1A) and *N. benthamiana* (Figure 1B). Particularly, after CymMV and ORSV infection, *p*PaAGO5b activity was significantly enhanced in *P. aphrodite* subsp. *formosana* and *N. benthamiana* leaves compared with no viral infection (Figure 1A,B). GUS activity levels were not detected in the mock treatment (infiltration with buffer only), negative control, or empty vector (EV; pKn). GUS activity results in *P. aphrodite* subsp. *formosana* and *N. benthamiana* revealed that only *p*PaAGO5b was induced during CymMV and ORSV infection.

### 2.3. Detection of pPaAGO5b Activity during Various Virus Infections and Viral Gene Expression in Transgenic N. benthamiana

**Figure 2:**
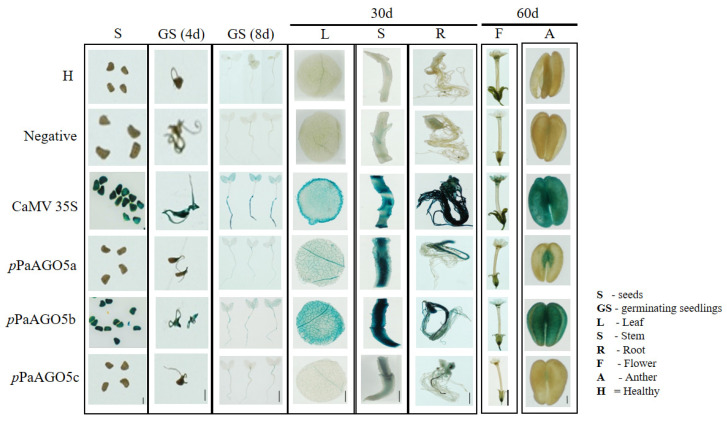
GUS histochemical staining in tissues of transgenic *N. benthamiana.* GUS expression driven by *p*PaAGO5a, *p*PaAGO5b, *p*PaAGO5c, CaMV 35S, and negative constructs. H represents healthy plants. As per the staining analysis, in CaMV 35S transgenic line, constitutive expression of GUS was observed in seeds, germinating seedlings, leaf, stem, root, flower, and anther. In the *p*PaAGO5a transgenic line, low expression of GUS was observed in germinating seedling, leaf, flower, and anther, moderate expression was observed in stem and root, and no expression was observed in seeds. In the *p*PaAGO5b transgenic line, higher expression of GUS expression was noticed in seeds, germinating seedlings, leaf, stem, root, flower, and anther. In the *p*PaAGO5c transgenic line, extremely low expression in germinating seedlings, leaf, and anther, low expression in the stem and root, and no expression in seeds have been observed. GUS activity was undetectable in healthy plants (H) and negative controls. The seeds, 4-and 8-day-old germinating seedlings, leaves, roots, stems of 30-day-old plants, and flowers and anthers of 60-day-old plants were incubated in GUS staining solution at 37 °C for 12 h. Scale bar of seeds: 0.1 cm; scale bar of anthers: 0.2 cm; the other scale bars: 1 cm.

Based on the preliminary GUS activity data (Figure 1A,B), we selected *p*PaAGO5b for further characterization. To investigate the strength of *p*PaAGO5b, infectious clones of various viruses were agroinfiltrated into transgenic *N. benthamiana* leaves harboring the reporter construct, pCAMBIA-*p*PaAGO5b::GUS. Before agroinfiltration, the T_3_ progeny per construct was confirmed by PCR to detect the presence of *GUS* and *HygR* (Supplementary Figure S4). We also explored tissue-specific GUS expression in transgenic *N. benthamiana* during the developmental stages by histochemical staining (Figure 2). Seeds, germinated seedlings, leaves, stems, roots, flowers, and anthers were collected from each line. According to the staining results (Figure 2), constitutive expression of GUS was observed in the *p*PaAGO5b transgenic line compared to those in *p*PaAGO5a and *p*PaAGO5c. In contrast, GUS activity was undetectable in the healthy plants and negative controls (Figure 2). These results indicate that PaAGO5b may play a significant role in plant growth and development.

**Figure 3:**
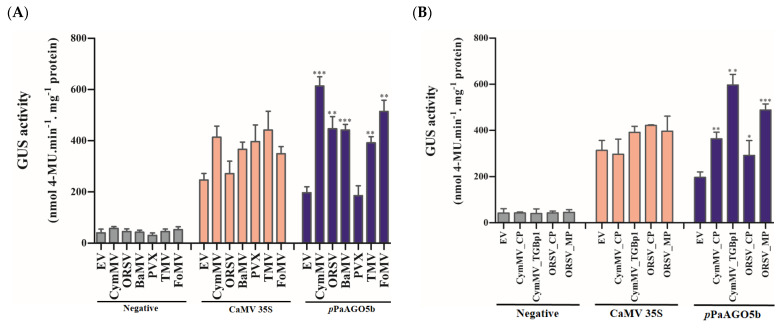
GUS fluorescent quantitative activity of *p*PaAGO5b during virus infection and expression of viral genes. (**A**) GUS fluorescent quantitative analysis of the negative CaMV 35S and *p*PaAGO5b during CymMV, ORSV, BaMV, PVX, TMV, and FoMV infection in transgenic *N. benthamiana* leaves at 3 dpi. (**B**) GUS fluorescent quantitative analysis of the negative CaMV 35S and *p*PaAGO5b during CymMV_CP, CymMV_TGBp1, ORSV_CP, and ORSV_MP overexpression in transgenic *N. benthamiana* leaves at 3 dpi. Each promoter GUS construct was assayed at least three times in four independent experiments. The GUS activity, 4-MU nmol-produced min^−1^ mg^−1^ protein, is represented as the mean ± SD of each negative control, CaMV 35S, *p*PaAGO5b. Data are mean ± SD, * p < 0.05, ** p < 0.01, *** p < 0.001 by Student’s *t*-test, respectively.

After confirming tissue-specific expression in T_3_ progeny, we quantified the GUS activity level driven by *p*PaAGO5b during CymMV and ORSV infection. As per GUS quantification, *p*PaAGO5b activity was significantly enhanced by 3.5-fold and 2.5-fold, respectively, compared to that without CymMV and ORSV infection (leaves infiltrated only with EV) (Figure 3A). To evaluate the compatibility of *p*PaAGO5b, we inoculated BaMV, PVX, TMV, and FoMV infectious clones into transgenic *N. benthamiana* leaves. GUS quantitative fluorescence results at 3 dpi showed that *p*PaAGO5b significantly increased the GUS activity during the infections of BaMV (2.5-fold), TMV (1.5-fold), and FoMV (2.5-fold), but not PVX (Figure 3A). In contrast, the GUS activity driven by *p*PaAGO5a and *p*PaAGO5c did not differ from those of CymMV, ORSV, BaMV, PVX, TMV, and FoMV (Supplementary Figure S5). Furthermore, GUS activity was not detected in negative controls (Figure 3A). These results suggest that, except for PVX, the remaining infiltrated virus infectious clones had an inducing effect on *p*PaAGO5b. However, we did not observe the inducing effect of PVX not only in the infiltration site but also in systemic leaves at 10 dpi (data not shown).

To determine the viral proteins involved in regulating *p*PaAGO5b activity, we infiltrated CymMV_CP, CymMV_TGBp1, ORSV_CP, and ORSV_MP overexpression constructs (Supplementary Figure S6) into transgenic *N. benthamiana* leaves. As a result, the *p*PaAGO5b activity was increased by 2-, 3.5-, 1-, and 2.5-fold with CymMV_CP, CymMV_TGBp1, ORSV_CP, and ORSV_MP overexpression, respectively, compared to those without viral gene expression (leaves infiltrated only with EV) (Figure 3B). However, *p*PaAGO5a and *p*PaAGO5c expressions were not significantly enhanced by the viral proteins we tested, as described (Supplementary Figure S7). In parallel, GUS activity was not detected in the negative controls (Figure 3B). These results suggest that CymMV_TGBp1 and ORSV_MP had a greater effect on *p*PaAGO5b.

### 2.4. Mapping of pPaAGO5b (Virus-Responsive Element) by Using 5′-Deletion Constructs in N. benthamiana during CymMV and ORSV Infection or Viral Gene Expression

**Figure 4:**
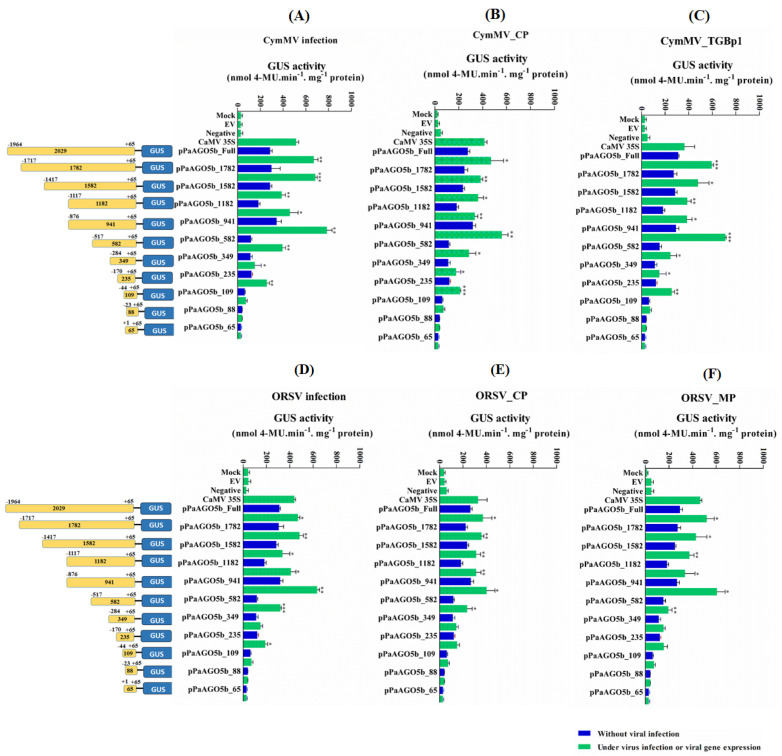
GUS fluorescent quantitative analysis of the 5′-deletion constructs of *p*PaAGO5b. The activity level of *p*PaAGO5b 5′-deletion constructs during (**A**) CymMV infection, (**B**) CymMV_CP. overexpression, (**C**) CymMV_TGBp1 overexpression, (**D**) ORSV infection, (**E**) ORSV_CP, and (**F**) ORSV_MP overexpression in wild-type *N. benthamiana* leaves at 3 dpi. Each 5′-deletion promoter construct (mentioned in the figure with length) was assayed at least three times in four independent experiments. The GUS activity, 4-MU nmol-produced min^−1^ mg^−1^ protein, is represented as the mean ± SD of each mock, empty vector (EV; pKn for the CymMV and ORSV infection; pEPYON-32K for the viral genes expression analysis), negative control and CaMV 35S, and 5′-deletion promoter GUS constructs. Data are mean ± SD, * p < 0.05, ** p < 0.01, *** p < 0.001 by Student *t*-test, respectively.

The *p*PaAGO5b showed significantly increased activity during viral infection and the overexpression of viral genes (Figure 3A,B). To determine which region of *p*PaAGO5b determines activity levels during viral infection, we functionally investigated the 5′-deletion constructs of *p*PaAGO5b based on *cis*-acting elements, which play a role during stress conditions. A total of ten 5′-truncated *p*PaAGO5b fragments of different sizes (−1782, −1582, −1182, −941, −582, −349, −235, −109, −88, and −65 bp upstream to TSS) were amplified from the *p*PaAGO5b full-length promoter. Each construct was co-infiltrated with CymMV or ORSV infectious clones or viral genes. The results of the 5′-deletion transient assay in *N. benthamiana* are shown in Figure 4. During CymMV infection (Figure 4A) or overexpression of CymMV_CP (Figure 4B) or CymMV_TGBp1 (Figure 4C), *p*PaAGO5b_941 exhibited maximal activity, followed by *p*PaAGO5b_full-length, *p*PaAGO5b_1782, *p*PaAGO5b_1582 as compared to those without CymMV infection or the expression of CymMV_CP or CymMV_TGBp1. During ORSV infection (Figure 4D) or overexpression of ORSV_CP (Figure 4E), ORSV_MP (Figure 4F) and *p*PaAGO5b_941 exhibited maximal activity, followed by *p*PaAGO5b_full-length, *p*PaAGO5b_1782, and *p*PaAGO5b_1582 as compared to those without ORSV infection or ORSV_CP or ORSV_MP. The 5′-deletion constructs *p*PaAGO5b_582, *p*PaAGO5b_349, *p*PaAGO5b_109, *p*PaAGO5b_88, and *p*PaAGO5b_65 (UTR alone) exhibited relatively poor activity compared with the remaining 5′-deletion constructs (Figure 4A–F). GUS activity levels were not detected in mock, EV, or negative control treatments.

### 2.5. Quantitative Analysis of TFs during Virus Infection and Yeast One-Hybrid Analysis

To examine the expression profiles of selected TFs identified in the in silico analysis (NbMYB94, NbREV8, NbLHY, NbMYB30, and NbCIR1), transgenic *N. benthamiana* leaves were infiltrated with CymMV infectious clones. The plants were kept at 25 °C with a 16 h light period, and leaf samples were collected at 72 h post infiltration (hpi). The real-time qRT-PCR results showed that among the five TFs, NbMYB30 and NbREV8 activity was significantly upregulated after CymMV infection compared with EV (pKn) infiltration (Supplementary Figure S8). Likewise, we analyzed the expression profile of TFs NbNAC42 and NbZFP3 and proved activator and repressor of NbAGO5 in *N. benthamiana*. The real-time qRT-PCR results showed that both NbNAC42 and NbZFP3 did not exhibit enhancement in activity (Supplementary Figure S8).

**Figure 5:**
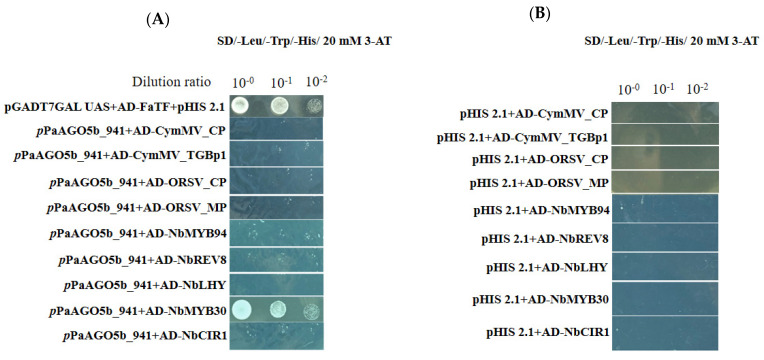
Physical interaction between *p*PaAGO5b_941 and viral genes or TFs in yeast cells. The full-length clones of CymMV_CP, CymMV_TGBp1, ORSV_CP, ORSV_MP, NbMYB94, NbREV8, NbLHY, NbMYB30, and NbCIR1 and were fused to pGADT74-AD and *p*PaAGO5b_941 fused to pHIS 2.1-BD were cotransformed and expressed in the yeast strain Y187. The transformed yeast cells were grown in non-selective media with histidine (SD/-Leu/-Trp) (Supplementary Figure S14) or (**A**) selective media without histidine with 20 mM 3-AT (SD/-Leu/-Trp/-His), followed by incubation at 30 °C for 3 days. The pHIS 2.1 vector and pGADT74-AD cotransformed with strawberry heat shock TF (FaTF) fused with pGBKT7 vector into yeast cells were used as the positive control. (**B**) The pHIS 2.1 vector and cotransformed with CymMV_CP, CymMV_TGBp1, ORSV_CP, ORSV_MP, NbMYB94, NbREV8, NbLHY, NbMYB30, and NbCIR1 into yeast cells were used as the negative controls.

To test whether the enhanced activity of *p*PaAGO5b was due to the direct effect of viral genes or an indirect effect through the interaction of TFs, we selected *p*PaAGO5b_941 and performed the Y1H assay. Based on the Y1H analysis, we did not find an interaction between *p*PaAGO5b_941 and viral genes such as CymMV_CP, CymMV_TGBp1, ORSV_CP, and ORSV_MP on SD/−Leu/−Trp/−His plates supplemented with 20 mM 3-AT (Figure 5A). Furthermore, we performed Y1H assay to examine the interaction between *p*PaAGO5b_941 and TFs. Among the five TFs (NbMYB94, NbREV8, NbLHY, NbMYB30, and NbCIR1), we observed the growth of *p*PaAGO5b_941 and NbMYB30 containing Y187 cells on SD/−Leu/−Trp/−His plates supplemented with 20 mM of 3-AT (Figure 5A). Therefore, we found that the enhanced activity of *p*PaAGO5b_941 during viral infection is indirect, indicating that virus infection could upregulate TF NbMYB30 and interact with the promoter region *p*PaAGO5b_941. We did not observe any physical interactions in the negative controls (Figure 5B). 

### 2.6. Effect of NbMYB30 on GUS Expression Driven by the pPaAG05b in N. benthamiana

**Figure 6:**
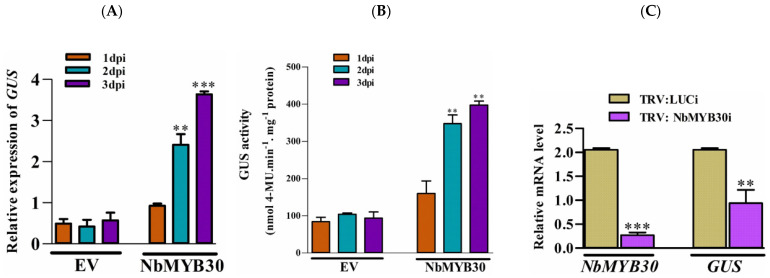
Effects of overexpression and silencing of NbMYB30 on *GUS* transcript levels in transgenic *N. benthamiana* plants. (**A**) The expression level of *GUS* was analyzed by real-time qRT-PCR, and (**B**) the activity level of GUS was analyzed by fluorimetric analysis. The transient expression effect of NbMYB30 significantly enhanced the GUS expression and activity level. (**C**) The TRV-based silencing of *NbMYB30* significantly downregulated the transcript level *GUS* in transgenic *N. benthamiana* plants. Data are mean ± SD, ** p < 0.01, *** p < 0.001 by Student *t*-test, respectively.

To validate the effect of NbMYB30 on *GUS* expression, driven by the *p*PaAGO5b, we performed the overexpression and TRV-based silencing experiments in transgenic *N. benthamiana* with pCAMBIA-*p*PaAGO5_941::GUS. To overexpress NbMYB30, the coding region of *NbMYB30* was amplified with a FLAG-tag, and its expression was confirmed by Western blotting (Supplementary Figure S9). After confirming the expression of NbMYB30, 28-day-old *N. benthamiana* leaves were agroinfiltrated with *A. tumefaciens* strain GV3850 cells harboring pEPFlag-NbMYB30. For analysis, the leaves were sampled at 1, 2, and 3 dpi. In addition, the expression level of *GUS* was analyzed by real-time qRT-PCR and GUS fluorometric analysis. The mRNA expression profile of *GUS* significantly increased during the overexpression of NbMYB30 compared with those infiltrated with EV (Figure 6A). Similar findings were observed through GUS fluorimetric analysis (Figure 6B). 

For loss-of-function experiment, *NbMYB30* expression was transiently knocked down using TRV-based VIGS. Plants infiltrated with the luciferase-silencing (Luci) construct were used as the negative control. Real-time qRT-PCR analysis displayed that the transcription of *NbMYB30* was specifically downregulated to 0.03-fold of that of the negative control at 10 dpi (Figure 6C). Furthermore, the expression level of *GUS* during *NbMYB30* knockdown decreased to 0.52-fold compared to that of the negative control (Figure 6C). The results of the overexpression and silencing analyses further support the assumption that *PaAGO5b* expression is positively associated with NbMYB30.

### 2.7. Responses of NbMYB30 on Challenges of CymMV and ORSV in N. benthamiana

To validate the effect of CymMV or ORSV infectious clones and viral genes on the expression of *NbMYB30*, we co-infiltrated the transgenic *N. benthamiana* plants with the pCAMBIA-*p*PaAGO5_941::GUS construct with infectious clones of CymMV, ORSV, and transient expression construct of viral genes. Real-time qRT-PCR expression profiles revealed that *NbMYB30* expression was significantly increased following the challenge with CymMV and ORSV, and expression of CymMV_CP, CymMV_TGBp1, ORSV_CP, and ORSV_MP compared with that in the EV-treated control plants (Supplementary Figure S10). These findings support that endogenous NbMYB30 significantly responded to viral infection and enhanced the activity levels. 

### 2.8. The Response of pPaAGO5b to Phytohormone Treatments

**Figure 7:**
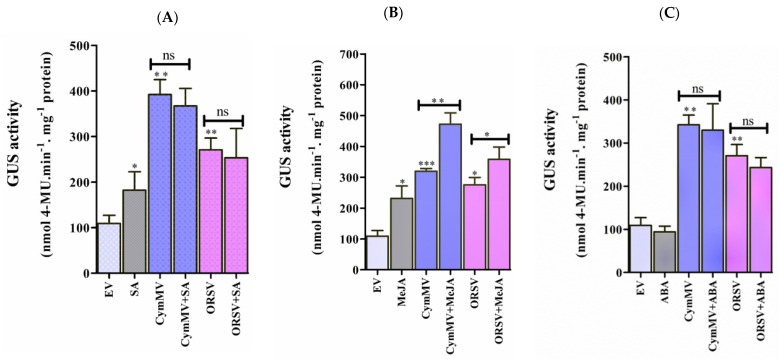

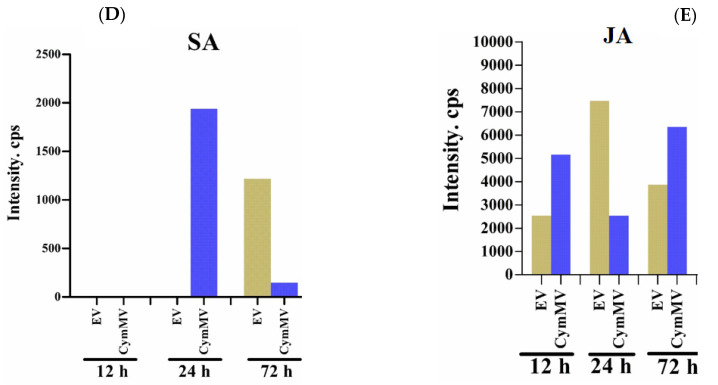
GUS fluorescent quantitative analysis during exogenous application of phytohormones and measurement of endogenous SA and JA levels. The activity of GUS driven by the *p*PaAGO5b measured during exogenous application of (**A**) 1 mM SA, (**B**) 100 µM MeJA, and (**C**) 100 µM ABA treatment alone or virus infection alone or a combination of phytohormones and virus infection in transgenic *N. benthamiana* leaves at 3 dpi. Effect of each hormone on GUS activity was assayed at least three times in four independent experiments. The GUS activity, 4-MU nmol-produced min^−1^ mg^−1^ protein, is represented as the mean ± SD. Data are mean ± SD, * p < 0.05, ** p < 0.01, *** p < 0.001 by Student *t*-test, respectively. ns = non-significant. (**D**) The content of endogenous SA and (**E**) JA levels in *N. benthamiana* during the infiltration of EV (pKn) and CymMV infection at 12, 24, and 72 hr. The intensity of SA and JA was measured by the ultra-high performance liquid chromatography-electrospray ionization tandem mass spectrometry (UHPLC-ESI-MS/MS) method.

Phytohormones, such as SA, JA, and ABA, play key roles during biotic stress. Various phytohormone-responsive elements were recognized in the promoter sequence of *p*PaAGO5b (Supplementary Table S2), indicating that *p*PaAGO5b may affect various phytohormone-related metabolic activities that regulate plant growth and development during pathogen infection. To evaluate the effect of phytohormones on *p*PaAGO5b, 1 mM SA, 100 µM MeJA, and 100 µM ABA were sprayed exogenously on transgenic *N. benthamiana* leaves. GUS fluorometric assays showed that GUS expression was significantly induced by SA (Figure 7A) and MeJA (Figure 7B) at 72 h, but not by ABA (Figure 7C) treatment. Furthermore, we examined the GUS activity levels with a combination of phytohormones and CymMV or ORSV infection at 72 h. According to GUS fluorimetric analysis, with the combination of virus infection and 1 mM SA or 100 µM ABA treatment, no significant increase was noted compared with CymMV and ORSV infection or hormone treatment alone (Figure 7A,C). However, *p*PaAGO5b activity significantly increased with the combination of CymMV or ORSV infection with 100 µM MeJA, compared with CymMV or ORSV infection or hormone alone (Figure 7B). These results indicate that SA and MeJA induced *GUS* driven by *p*PaAGO5b. Furthermore, the combination of virus infection and MeJA significantly induced *GUS*, specifying that MeJA might act as a major inducing hormone during CymMV or ORSV infection. This requires further investigation. 

Consequently, we examined the expression profile of *N. benthamiana* nonexpressor of pathogenesis-related genes-1 (*NbNPR1*) (SA marker gene), allene oxide synthase 2 (*NbAOS2*)*,* lipoxygenase 2 (*NbLOX2*) (JA biosynthesis genes), 9-cis-epoxycarotenoid dioxygenase *NbNCED3,* and zeaxanthin epoxidase (*NbZEP*) (ABA biosynthesis genes) during exogenous application of SA (1 mM), MeJA (100 µM), and ABA (100 µM) at 24 hpi. Real-time qRT-PCR results showed that *NbNPR1*, *NbAOS2,* and *NbLOX2* were significantly upregulated, whereas *NbNCED3* and *NbZEP* were not upregulated (Supplementary Figure S11). This indicates that the exogenous application of SA and MeJA significantly upregulated and induced *p*PaAGO5b_941, which may be due to SA-and MeJA-responsive elements (Supplementary Figure S12).

To measure endogenous SA and JA levels, *N. benthamiana* leaves were vacuum-infiltrated with EV (pKn) and CymMV infectious clones. After infiltration, leaf samples were collected at 12, 24, and 72 h, and SA and JA contents were measured using UHPLC-ESI-MS/MS. The intensity of SA was not detected in EV vector-treated leaves at 12 and 24 h, but it significantly increased at 72 h compared with CymMV-treated leaves. However, at 24 h, the intensity of SA was significantly higher in the CymMV-treated leaves than in the EV leaves (Figure 7D). In addition, the JA intensity was significantly enhanced at 12 and 72 h in CymMV-treated leaves compared with EV-treated leaves. However, at 24 h, JA intensity was significantly decreased in CymMV-treated leaves compared to EV-treated leaves (Figure 7E). We hypothesized that SA and JA act antagonistically during CymMV infection based on this endogenous measurement of SA and JA. Further experiments are needed to analyze the underlying molecular mechanism of action between SA and JA during CymMV infection. 

### 2.9. Effect of PaMYB30 on PaAGO5b Expression and Accumulation of CymMV in P. aphrodite subsp. formosana

**Figure 8:**
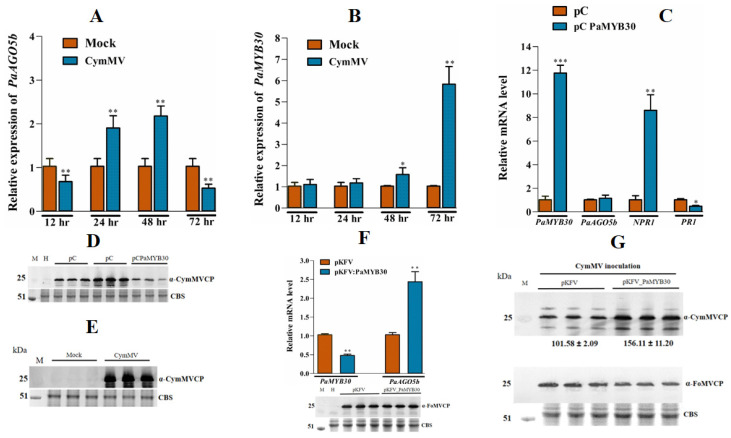
Expression profile of *PaAGO5b*, *PaMYB30,* and CymMV accumulation in CymMV infected leaves. (**A**) Expression profile of *PaAGO5b* and (**B**) *PaMYB30* in the *P. aphrodite* subsp. *formosana* leaves infiltrated with mock (pKn) or CymMV infectious clone. (**C**) The transcript accumulation of *PaAGO5b*, *PaNPR1,* and *PaPR1* during PaMYB30 overexpression. For the PaMYB30 overexpression analysis, *P. aphrodite* subsp. *formosana* leaves were infiltrated with empty vector pCambia-UbI1-ZsGFP vector (pC) or PaMYB30 expression vector pC PaMYB30. (**D**) The accumulation of CymMV in CymMV infected leaves at 72 hpi was analyzed by Western blot. (**E**) The accumulation of CymMV during PaMYB30 overexpression leaves at 72 hpi was analyzed by Western blot. (**F**) *PaMYB30* and *PaAGO5b* transcript accumulation during PaMYB30 silencing at 10 dpi was analyzed using real-time qRT-PCR. (**G**) The CymMV accumulation in the *PaMYB30*-silenced leaves. The PaMYB30 silenced leaves were further agroinfiltrated with CymMV infectious clone at 10 dpi (days post infiltration of agrobacterium EHA105 harboring pKFV or pKFV-MYB30 vector). The leaves were collected at 15 dpi, and the CymMV accumulation in the leaves was analyzed using Western blot. The FoMV accumulation in the leaves was analyzed using Western blot. For real-time qRT-PCR, the expression levels of each transcript, presented as normalized fold changes relative to that from mock-inoculated leaves (Mock) or EV are shown. Values are means ± SD of three biological replicates. Data are mean ± SD, * p < 0.05, ** p < 0.01, *** p < 0.001 by Student *t*-test, respectively. For Western blot, the accumulation of FoMV and CymMV were detected by respective specific anti-serum, α-FoMV CP and α-CymMV CP. Rubisco was stained with Coomassie-brilliant-blue and shown as a loading control.

In silico analysis of protein similarity of NbMYB30 in Orchidstra 2.0 [34] revealed that *P. aphrodite* subsp. *formosana* MYB30 (PaMYB30) (PATC157886) shares 52% protein similarity with NbMYB30, making PaMYB30 the closest relative of NbMYB30 in orchids. Therefore, we speculated that PaMYB30 is a candidate that responds to CymMV infection and activates the expression of endogenous PaAGO5b, as observed in *N. benthamiana* leaves. To verify this hypothesis, we inoculated *P. aphrodite* subsp. *formosana* leaves with a CymMV infectious clone via agro-infiltration and collected leaves to analyze expression profiles of *PaMYB30* and *PaAGO5b* at 12, 24, 48, and 72 h post-inoculation (hpi). Real-time qRT-PCR analysis indicated that the accumulation of *PaAGO5b* transcript was significantly elevated at 48 and 72 hpi (Figure 8A), as observed previously [33]. However, the accumulation of *PaMYB30* transcript increased in inoculated leaves at 24 and 48 hpi but decreased at 12 and 72 hpi (Figure 8B). The fluctuation of PaMYB30 occurred at least 24 h earlier than that of PaAGO5b after CymMV inoculation, suggesting that PaMYB30 could respond to virus infection.

Next, we investigated the causation between PaMYB30 and PaAGO5b expression. We overexpressed FLAG-tagged PaMYB30 and a PVY silencing suppressor, HC-pro, used to improve protein expression in CymMV-inoculated *P. aphrodite* subsp. *formosana* leaves. We found that the overexpression of PaMYB30 did not alter the expression level of PaAGO5b (Figure 8C) but unexpectedly resulted in a reduction in CymMV accumulation (Figure 8E). Before analyzing CymMV accumulation during PaMYB30 overexpression, we confirmed CymMV expression in *P. aphrodite* subsp. *formosana* leaves (Figure 8D). To verify these results, *PaMYB30* was silenced using the VIGS vector pKFV [35]. Real-time qRT-PCR analysis showed that *PaMYB30* expression was efficiently silenced by VIGS (Figure 8F); however, *PaAGO5b* expression was significantly upregulated (Figure 8F). To clarify the role of *PaMYB30* in plant defense systems, PaMYB30-silenced and mock-inoculated leaves (agroinfiltrated with empty VIGS vector, pKFV) were inoculated with CymMV. After quantifying viral accumulation, we found that PaMYB30 knockdown contributed to a higher accumulation of CymMV (Figure 8G).

### 2.10. Effects of NbMYB30, NbNAC42, and NbZFP3 Expression on the Accumulation of CymMV in P. aphrodite subsp. formosana

To verify the function of NbMYB30 in CymMV accumulation in *P. aphrodite* subsp. *formosana*, we overexpressed FLAG-tagged NbMYB30 in *P. aphrodite* subsp. *formosana* and evaluated its effect on CymMV accumulation. Moreover, our previous experiments demonstrated that the TFs NbNAC42 and NbZFP3 might participate in the activation of NbAGO5 expression during BaMV infection (provisionally accepted). Thus, these two TFs were also expressed in *P. aphrodite* subsp. *formosana* and subjected to the same evaluation as NbMYB30. For CymMV accumulation, a slight reduction was shown in NbMYB30 expressing leaves (Supplementary Figure S13); however, 2.4- and 2.6-fold increases were observed in NbNAC42- and NbZFP3-expressing leaves (Supplementary Figure S13). These results demonstrated that NbMYB30 physically interacts with the PaAGO5b promoter and participates in specific resistance mechanisms in *P. aphrodite* subsp. *formosana*. However, the NbNAC42 and NbZFP3 transcription factors may not function properly in exogenous environments. 

## 3. Discussion

Transcription regulation is a crucial process that is affected by gene promoters. Appropriate studies on promoter activity during viral infection have not been found for plants, except for a few studies [21,36]. Recently, we identified the induced activity of NbAGO5 through the binding of TF NbNAC42 and repressed the activity by the binding of TF NbZFP3 (provisionally accepted). Promoter sequence analysis of NbAGO5 and *p*PaAGO5b shows that the sequence identity between these two promoters is around 30% only. Moreover, presence of *cis*-acting element in the promoter region is also diverse. Experimental findings from *Arabidopsis*, rice, *N. benthamiana,* and orchids showed that AGOs play a key role in antiviral defense mechanisms. The results of this study revealed the effect of viruses on AGO promoter activity through the analysis of *p*PaAGO5b. 

CymMV and ORSV contain monopartite, positive-sense RNA genomes, and are the most widespread and economically significant viruses among 50 orchid-infecting viruses [32]. In addition to their natural hosts, CymMV and ORSV can infect *N*. *benthamiana*, frequently used as a systemic host in plant virus research [37]. Owing to the slow growth of *P. aphrodite* subsp. *formosana*, we performed experiments on *N*. *benthamiana* to test the activity of the *p*PaAGO5s constructs fused with GUS. To interpret the differential expression patterns of GUS driven by *p*PaAGO5a, *p*PaAGO5b, and *p*PaAGO5c, we compared the promoter sequences (Supplementary Tables S1–S3). Preliminary GUS activity analysis showed that *p*PaAGO5b was significantly enhanced during CymMV and ORSV infection compared to *p*PaAGO5a and *p*PaAGO5c (Figure 1A,B). These results are consistent with those of previous studies [33]. The supported results from PaAGO5b expression [37] and current *p*PaAGO5b fluorescent quantitative analyses suggest that PaAGO5b may serve as the first layer of the immune system during viral infection. The highest activity of *p*PaAGO5b may be due to the presence of some critical variances in the spreading of putative *cis*-acting elements, such as ABRE, TGACG motif, and TGA- and TCA-elements, which are absent in *p*PaAGO5c, or GARE-motif and TCA-elements, which are absent in *p*PaAGO5a (Supplementary Tables S1–S3). The tissue-specific expression profile revealed that the higher expression of GUS driven by *p*PaAGO5b (Figure 2) indicates that PaAGO5b may play a key role during plant growth and development. However, the roles of *p*PaAGO5a and *p*PaAGO5c in plant growth and development need to be explored. 

Early enhanced activity of *p*PaAGO5b was observed during CymMV, ORSV, BaMV, TMV, and FoMV, but not PVX infection in *N. benthamiana* (Figure 3A). In contrast, *Arabidopsis* AGO5 expression was induced by PVX infection. In particular, AtAGO2 and AtAGO5 are required to fully restrict PVX infection in systemic tissues [7]. This finding may be due to the different host–factor interactions during PVX infection. *p*PaAGO5b activity was significantly increased by the overexpression of CymMV_CP, CymMV_TGBp1, ORSV_CP, and ORSV_MP (Figure 3B). Principally, GUS activity was significantly enhanced during CymMV infection, and the expression of CymMV_TGBp1 and ORSV_MP (Figure 3A,B). This finding may be due to CymMV accumulation at the initial to middle stages of infection, which increases the activity of *p*PaAGO5b. Previous studies have shown that PaAGO5b expression is significantly elevated when *P. aphrodite* subsp. *formosana* plants are singly infected with CymMV [33]. The higher activities of CymMV_TGBp1 and ORSV_MP on *p*PaAGO5b indicate that these two viral proteins are crucial for inducing *p*PaAGO5b. However, there was no direct proof that CymMV_TGBp1 or ORSV_MP was involved in suppressing RNA silencing. This indicates that PaAGO5b may act as a lead protein in controlling viral infections. We demonstrated a significant increment in the expression of *NbAGO5* during BaMV_TGBp1 overexpression (provisionally accepted). BaMV and CymMV belong to the genus *potexvirus*, and TGBp1 may act as a major inducer of AGOs during potexvirus infection. 

From the early induced activity of *p*PaAGO5b, we further mapped the virus-responsive element of *p*PaAGO5b toward viral infection. Transient expression analysis revealed that the 5′-deletion construct, *p*PaAGO5b_941, showed significantly higher activity toward CymMV (Figure 4A), ORSV infection (Figure 4D), and the expression of viral genes such as CymMV_CP (Figure 4B), CymMV_TGBp1 (Figure 4C), ORSV_CP (Figure 4E), and ORSV_MP (Figure 4F). The higher activity of *p*PaAGO5b_941 may be due to the core-and stress-related *cis*-acting elements within this region (Supplementary Table S2). 

Previous studies of virus infection on plant AGO genes did not distinguish a direct or indirect virus-induced effect. To explore this, we cloned the viral genes for Y1H analysis. Based on Y1H analysis, we found that viral proteins did not directly interact with *p*PaAGO5b_941 (Figure 5A). Furthermore, screened TFs expression profiles were analyzed, and the results showed that NbREV8 and NbMYB30 were significantly enhanced during CymMV infection (Supplementary Figure S8); however, YIH analysis showed that only NbMYB30 physically interacted with *p*PaAGO5b_941 (Figure 5A). NbREV8 may interact with other AGOs or pathogen-related genes during viral infections. However, there are no prior studies on the role of the NbMYB30 TF in AGO gene expression during viral infection. Real-time qRT-PCR and Y1H analysis revealed that NbMYB30 might be upregulated during viral infection and directly binds to the region of *p*PaAGO5b_941 to regulate PaAGO5b expression. The putative MYB-binding site AACAAA reported to be targeted by MYB30 [38] was found at position 584 in *p*PaAGO5b_941. It is possible that the NbMYB30 TF binds to the AACAAA site to induce PaAGO5b at the time of infection. Furthermore, NbMYB30 overexpression (Figure 6A) and VIGS analysis (Figure 6C) in *N. benthamiana* revealed that NbMYB30 positively regulated *p*PaAGO5b in response to viral infections.

NbMYB30 belongs to the R2R3 MYB TF and is part of the MYB TF family. The MYB TF family plays a significant role in plant immune responses to biotic and abiotic stresses [39,40,41,42]. Published research findings on MYB TFs involved in the defense response to viral invasion are currently limited. However, inducible NtMYB1 has been identified in tobacco during TMV infection [25]. Tomato MYB28 TF (SlMYB28), and an R2R3-MYB TF expression level was strongly induced by *Tomato yellow leaf curl virus* (TYLCV) infection in tomato [26]. In addition, upregulation of AtMYB96 has been reported to be induced upon *Cauliflower mosaic virus* gene VI (P6) infection [43] in *Arabidopsis*. A total of 18 differentially expressed MYB TFs have been identified in watermelon during the invasion of *Cucumber green mottle mosaic virus* (CGMMV) [24]. Among them, 15 and 3 MYB TFs were up- and downregulated, respectively, in the leaf tissues of watermelon. These published results and current findings emphasize the role of MYB TF family proteins during virus infection.

The plant hormones SA, JA, and ABA, play key roles in helping plants balance their responses to viral stress [16,17,18,44]. Owing to hormone-responsive elements, *p*PaAGO5b_941 (Supplementary Figure S12) may affect various phytohormone-related metabolic activities during viral stress. We evaluated the effect of exogenous application of SA (1 mM), MeJA (100 µM), and ABA (100 µM) in *N. benthamiana*. GUS activity driven by *p*PaAGO5b_941 was significantly upregulated after exogenous application of SA (Figure 7A) and MeJA (Figure 7B) but not ABA (Figure 7C). The expression profile of *NbNPR1*, *NbAOS2, NbLOX2*, *NbNCED3,* and *NbZEP* showed that *NbNPR1*, *NbAOS2,* and *NbLOX2* were significantly upregulated, whereas *NbNCED3* and *NbZEP* were not enhanced (Supplementary Figure S11). This indicates that the exogenous application of SA and MeJA significantly upregulated and induced *p*PaAGO5b_941, which may be due to SA-and MeJA-responsive elements (Supplementary Figure S12). Furthermore, we assumed that SA and JA have a possible role in defense mechanisms during viral infection. We noticed that the combination of MeJA and CymMV or ORSV increased the activity of GUS when compared with the exogenous application of MeJA or virus infection alone (Figure 7B). Similar GUS activities were not observed with the application of SA or ABA in combination with CymMV and ORSV (Figure 7A,C). The significant increase in the activity of *p*PaAGO5b_941 after MeJA application during viral infection indicates that MeJA acts as a major hormone in viral defense mechanisms. However, SA application alone increased the *p*PaAGO5b_941 activity level (Figure 7A) compared to the combination of virus infection, which needs to be further explored. 

To validate the antagonistic relationship, endogenous SA and JA levels were measured. In *N. benthamiana,* the CymMV-inoculated experiments revealed that the accumulation of SA and JA was typically antagonized (Figure 7D,E), as reported in previous studies [45]. The SA level at 12 hpi in the EV- or CymMV-inoculated leaves was lower than the detection limit of our mass spectrometer (Figure 7D). The SA level at 24 hpi in EV-inoculated leaves remained undetectable but could be detected in CymMV-inoculated leaves at 24 hpi (Figure 7D). However, when comparing the JA level in CymMV-inoculated leaves to that in EV-inoculated leaves, it was higher at 12 hpi but lower at 24 hpi (Figure 7E). However, the SA and MeJA signaling pathways sometimes affect each other through a complex network of synergistic and antagonistic interactions [46,47]. Even though the TCA-element and TGACG motifs are present at the core promoter region of *p*PaAGO5b_941 (Supplementary Figure S12), the enhanced GUS activity under MeJA indicates its significance during virus infection. A few studies have revealed that regulatory networks enhance RNA-silencing activity via JA signaling. For instance, in rice, OsAGO18 promotes antiviral defense against RSV via transcriptional activation by jasmonate signaling [21]. The exogenous supply of MeJA efficiently reduces RBSDV infection in rice, whereas inhibition of the JA response enhances RBSDV infection [48,49]. Further investigation is required to investigate the crosstalk between SA and JA and the underlying molecular mechanism of these hormones on PaAGO5b activity during viral infection. Although we did not perform hormone analyses on *P. aphrodite* subsp. *formosana*, it was evident that PaMYB30 was suppressed at 12 hpi and activated at 24 hpi (Figure 8B).

This study revealed that the expression profiles of PaMYB30 and PaAGO5b are negatively correlated during CymMV infection. Considering that a distinct physical interaction between NbMYB30 and *p*PaAGO5b_941 was demonstrated (Figure 5A), the exogenous expression of NbMYB30 served as an activator or *p*PaAGO5b_941. Therefore, we cannot exclude the possibility that PaAGO5b expression is controlled by a potential suppressor element that lies outside the *p*PaAGO5b_941 region or the interaction of PaMYB30 with the potentially endogenous regulator in *P. aphrodite* subsp. *formosana*. Thus, PaAGO5b expression could only be activated when PaMYB30 levels decreased (Figure 8F). However, CymMV accumulation was significantly lower with PaMYB30 overexpression (Figure 8E) and higher with PaMYB30 silencing (Figure 8G). Although PaMYB30 has a significant defense role during viral infection in *P. aphrodite* subsp. *formosana*, with PaMYB30 expression, the nature of the antiviral response in orchid under PaMYB30 regulation remains unclear. The *P. aphrodite* subsp. *formosana* *NPR1* and *PR1* expression levels were significantly up- and downregulated, respectively, with the overexpression of PaMYB30 (Figure 8C), indicating PaMYB30 may cast regulatory antiviral activity through NPR1/PR1-related pathway. NbMYB30 overexpression in *P. aphrodite* subsp. *formosana* resulted in a slight decrease in CymMV accumulation (Supplementary Figure S13) compared with NbNAC42 and NbZFP3, a proven activator and repressor of NbAGO5 (provisionally accepted), indicating that NbMYB30 acts as an activator of PaAGO5b during viral infection. Our findings provide further insight into the role of PaMYB30 during virus infection and the defense response upon orchid–virus interactions. 

**Figure 9:**
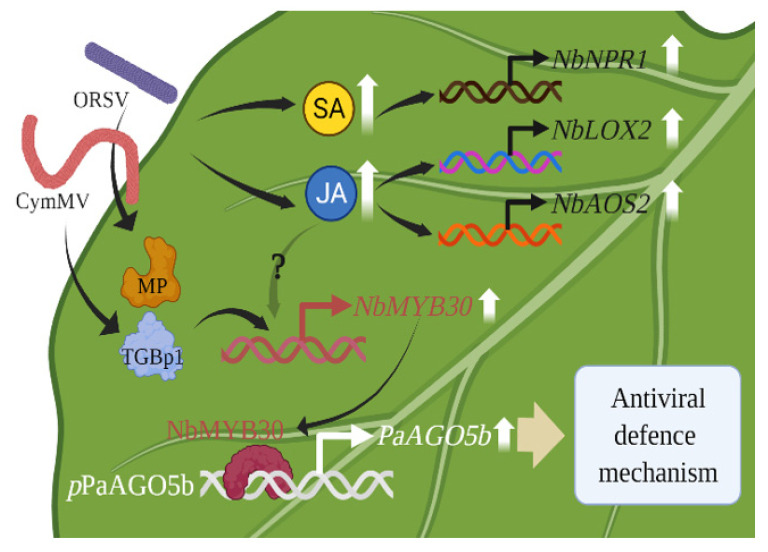
A model for enhancing antiviral defense mechanism through the activation of *p*PaAGO5b. The CymMV or ORSV infectious clones and their TGBp1, a CymMV-encoded protein, and MP, an ORSV-encoded protein, may upregulate plant defense-related TF NbMYB30. The NbMYB30 TF binds to the *p*PaAGO5b (*p*-indicates the promoter) and transcriptionally activates PaAGO5b expression to enhance the antiviral defense mechanism. During virus infection, phytohormones SA and JA and their marker genes were upregulated. Principally, JA was significantly enhanced; however, upregulation of NbMYB30 under JA enhancement during virus infection has not yet been explored. *LOX2*—lipoxygenase 2; *AOS2*—allene oxide synthase 2, *NPR1*—nonexpressor of pathogenesis-related genes-1.

In the current study, we characterized and functionally validated *p*PaAGO5b in *N. benthamiana* during viral infection (Figure 9).

GUS fluorescence analysis of stably transformed *N. benthamiana* leaves revealed that *p*PaAGO5b was induced during CymMV and ORSV infection and overexpression of viral genes; notably, during CymMV, CymMV_TGBp1, and ORSV_MP. The 5′-deletion fragment analysis showed that *p*PaAGO5b_941 had a higher inducible activity. Y1H analysis showed that viral infection indirectly induced the activity of *p*PaAGO5b_941 through the interaction of NbMYB30 TF, an activator of *p*PaAGO5b_941. An exogenous supply of SA, MeJA, and ABA in transgenic *N. benthamiana* showed that SA and MeJA induced *p*PaAGO5b_941. However, the combination of phytohormones and CymMV and ORSV infection revealed that MeJA was significantly enhanced, indicating that JA is the primary hormone inducing *p*PaAGO5b_941 during viral infection. This can help understand the molecular mechanism of PaAGO5b in RNA silencing during virus invasion. Furthermore, the thorough understanding of the regulatory mechanism may benefit the development and application of *p*PaAGO5b as an inducible promoter in the future.

## 4. Materials and Methods

### 4.1. Cloning and In Silico Exploration of the PaAGO5s Promoter 

Genomic DNA from *P. aphrodite* subsp. *formosana* leaves was extracted using the CTAB method [50], and used as a template for cloning promoter sequences. The primers (Supplementary Table S4) used for amplifying the putative promoter sequences of PaAGO5a, PaAGO5b, and PaAGO5c were designed from the translational start site, ATG, to about 2000 nucleotides upstream of each gene. After PCR amplification, the positive DNA fragments of PaAGO5a, PaAGO5b, and PaAGO5c were purified and separately ligated into a T&A cloning vector (YEASTERN Biotech Co., Ltd., Taipei City, Taiwan). The cloned fragments were sequenced and confirmed using the BLAST search against the Orchidstra 2.0 database [34]. Furthermore, the transcription start sites (TSS) of the PaAGO5a, PaAGO5b, and PaAGO5c promoters were confirmed by 5′-rapid amplification of cDNA ends (5′-RACE, Takara Bio, San Jose, CA, USA). 

To predict the *cis*-acting regulatory elements present in the cloned PaAGO5s promoter region, we used in silico analysis in PlantCARE (http://bioinformatics.psb.ugent.be/webtools/plantcare/html/ accessed on 20 June 2022) [51]. 

### 4.2. Construction of pPaAO5s::GUS Fusion Vectors

A 2759-bp *Hind*III-*Nco*I fragment containing *p*PaAGO5a, a 2029-bp *Xba*I-*Nco*I fragment containing *p*PaAGO5b, and a 2589-bp *Pst*I-*Nco*I fragment containing *p*PaAGO5c were sliced from the T&A cloning vector and subcloned into the same sites as the binary vector pCAMBIA1305.2 (Marker Gene Technologies, Eugene, OR, USA) after enzymatic digestion to replace the CaMV 35S promoter, which controls the expression of the GUS plus gene. The resulting constructs were named pCAMBIA-*p*PaAGO5a::GUS, pCAMBIA-*p*PaAGO5b::GUS, and pCAMBIA-*p*PaAGO5c::GUS, respectively. The vector pCAMBIA1305.2 with the CaMV 35S promoter was used as a positive control (CaMV 35S). The promoter-less vector pCAMBIA1391Z was used as the negative control. 

### 4.3. In Planta Agrobacterium-Mediated Transformation of N. benthamiana Plants

The constructed pCAMBIA-*p*PaAGO5a::GUS, pCAMBIA-*p*PaAGO5b::GUS, and pCAMBIA-*p*PaAGO5c::GUS plasmids were introduced into the *Agrobacterium tumefaciens* culture GV3850 individually by electroporation [52]. *A. tumefaciens*-mediated transformation procedures were performed as previously described [53]. Transformants were regenerated using the method described [54]. T_0_ positive transformants were screened for *hygromycin* (*Hyg*R) resistance [55]. The T_1_ and T_2_ positive transformants were confirmed by PCR amplification (primers used are listed in Supplementary Table S5) and GUS staining and grown to generate T_3_ transgenic plants. Homozygous transgenic lines of the T_3_ generation were chosen for subsequent investigation via segregation ratio analysis.

### 4.4. Construction of pPaAGO5b Deletion Promoters::GUS Fusion Vectors

Ten 5′-truncated *p*PaAGO5b fragments of different sizes (−1782, −1582, −1182, −941, −582, −349, −235, −109, −88, and −65 bp to −1 bp) were amplified from the *p*PaAGO5b full-length promoter and subcloned into the *Xba*I-*Nco*I sites of pCAMBIA1305.2 by replacing the original CaMV 35S promoter (primers used are listed in Supplementary Table S6). 

### 4.5. Construction of Virus Gene Plasmids and Agroinfiltration of Virus Infectious Clones and Viral Genes into Transgenic N. benthamiana

Different infectious clones, including CymMV [35], ORSV [35], BaMV [56], PVX [57], TMV, and *Foxtail millet mosaic virus* (FoMV) [35] used in this study were mainly based on the backbone vector pKn [58]. Therefore, for the overexpression of viral genes such as CymMV coat protein (CymMV_CP; 672 bp), CymMV triple gene block protein 1 (CymMV_TGBp1; 702 bp), ORSV coat protein (ORSV_CP; 477 bp), and ORSV movement protein (ORSV_MP; 840 bp), the corresponding coding regions were amplified from the CymMV and ORSV full-length infectious clones and cloned into the plasmid pEPYON-32K [59] with the CaMV 35S promoter. The primers used for cloning viral genes are listed in Supplementary Table S7. 

To analyze the stress responses caused by viruses and viral genes, *A. tumefaciens* strain GV3850 cells harboring infectious clones of CymMV, ORSV, BaMV, PVX, TMV, FoMV, and viral gene expression constructs were agroinfiltrated into *N. benthamiana* plants [15]. The *A. tumefaciens* cultures were collected by centrifugation, resuspended in infiltration buffer (10 mM MES buffer, pH 5.5, and 10 mM MgCl_2_), adjusted to OD_600_ = 0.5, and agroinfiltrated into the leaves of each test plant using a needleless syringe. The leaves were harvested three days post-inoculation (dpi) and subjected to the GUS activity assay.

### 4.6. GUS Histochemical Staining and Fluorescent Quantitative Assay

The GUS staining and activity of infiltrated constructs were estimated by the standard method [60]. The fluorescence was measured at excitation and emission wavelengths of 365 nm and 455 nm using a fluorimeter (SpectraMax M2, San Jose, CA, USA). GUS activity was calculated as nmol 4-methylumbelliferone (4-MU)-generated min^−1^ mg^−1^ protein.

### 4.7. Identification of Transcription Factors (TFs) and Yeast One-Hybrid (Y1H) Analysis

PlantPan 3.0 (http://plantpan.itps.ncku.edu.tw/ accessed on 20 June 2022) [61] was used to identify co-expressed TFs with the PaAGO5b promoter. By selecting hormone treatment conditions, we obtained a list of TFs from *Arabidopsis* that were co-expressed with the queried promoter sequence, as indicated by a Pearson correlation coefficient with a *p*-value > 0.9. To obtain a short list of TFs regulated only by ABA, MeJA, and SA, gene ontology and functional descriptions of these TFs were queried manually. Finally, a total of five TFs (MYB94, REV8, late elongated hypocotyl, and circadian clock associated-1-like protein 1, MYB30, and Circadian 1 (CIR1) transcription factor) were selected from *Arabidopsis*, and their full-length nucleotide sequences were used as a template to retrieve the TFs sequences (Supplementary Table S8) from *N. benthamiana* using draft genome sequence database (https://solgenomics.net/organism/Nicotiana_benthamiana/genome accessed on 20 June 2022).

To determine the interaction between *p*PaAGO5b and viral genes or TFs, we performed Y1H assay. The *p*PaAGO5b_941 sequence was cloned into the yeast reporter vector pHIS2.1-BD (Clontech Laboratories, Inc., San Jose, CA, USA) after digestion with the *Sac*I and *EcoR*I enzymes. Full-length coding regions of the viral genes and TFs were amplified and cloned into the pGADT7-AD vector (Clontech Laboratories, Inc., CA, USA). After confirming the sequence orientation of the cloned viral genes and TFs, the pHIS2.1 vector containing the *p*PaAGO5b_941 was cotransformed individually with the pGADT7-viral genes and TFs into *Saccharomyces cerevisiae* strain Y187. Serial dilution transformant growth assays evaluated Protein-DNA interactions on SD/−Leu/−Trp/−His plates supplemented with 20 mM 3-AT. The primers used for the TFs amplification are listed in Supplementary Table S9. 

### 4.8. Transient Expression and Virus-Induced Gene Silencing (VIGS) of NbMYB30 

To overexpress FLAG-NbMYB30 in *N. benthamiana*, plasmid pEPFlag-NbMYB30 was generated. The coding sequence (CDS) for *NbMYB30* (1083 bp) was amplified from *N. benthamiana* cDNA using PCR (primers used are listed in Supplementary Table S10). The PCR product was gel-purified, digested with *Pst*I and *Sac*I, and used to replace the corresponding fragments in pEP-mGFP [59]. For overexpression, pEPFlag-NbMYB30 was introduced into *A. tumefaciens* strain GV3850 by electroporation. *A. tumefaciens* cultures were collected by centrifugation and resuspended in an infiltration buffer. Suspensions were adjusted to OD_600_ = 0.5 and infiltrated by a needleless syringe into the leaves of each test plant. 

*Tobacco rattle virus* (TRV)-based VIGS was used to knockdown the expression of *NbMYB30*. A 240-bp fragment of the *NbMYB30* 3′ untranslated region (UTR) sequence was amplified by PCR using *N. benthamiana* cDNA as a template (primers used are listed in Supplementary Table S10). The amplified PCR product was gel-purified, digested with *Eco*RI and *Bam*HI, and cloned into the pTRV2 plasmid [62] to generate pTRV2-NbMYB30. The pTRV1- and pTRV2-based constructs were electroporated into the *A. tumefaciens* strain C58C1 for knockdown experiments, as previously described [57].

### 4.9. Agroinfiltration and Virus Inoculation in P. aphrodite subsp. formosana

Agroinfiltration of *P. aphrodite* subsp. *formosana* leaves were performed as reported by [33]. Briefly, pCAMBIA-Ubi1-ZsGFP, pCAMBIA-Ubi1-PaMYB30, and CymMV infectious constructs were electroporated into *A. tumefaciens* strain EHA105. Aliquots of a 2 mL saturated culture of agrobacteria were poured into an 18 mL LB medium containing ampicillin and kanamycin and incubated at 28 °C for 3 h. Bacteria were then pelleted by centrifugation and incubated in AB-MES buffer with constant shaking (60 rpm) at 28 °C for 24 h. Finally, the bacteria were pelleted by centrifugation and resuspended in AB-MES+1/2 MS infiltration buffer containing 200 µM acetosyringone. The expression construct inoculum was adjusted to give an OD_600_ of 10. The inoculum containing the CymMV infectious clone was adjusted to an OD_600_ value of 0.5. The coding sequence (CDS) for *PaMYB30* (912 bp) was amplified from *P. aphrodite* subsp. *formosana* cDNA using PCR (primers used are listed in Supplementary Table S11). 

### 4.10. PaMYB30 Gene VIGS Construct, pKFV_PaMYB30

The VIGS construct for the knockdown of *PaMYB30* in *P. aphrodite* subsp. *formosana* was constructed as previously reported [33]. Briefly, the 241 bp fragment covering the CDS and 3′ UTR regions used for silencing *PaMYB30* was flanked by the *Hpa*I restriction enzyme cut site through PCR amplification. After *HpaI* digestion, the fragment was ligated with *Hpa*I digested pKFV [35] to generate pKFV-PaMYB30. The primers used for the pKFV-PaMYB30 VIGS construct are listed in Supplementary Table S11. 

### 4.11. RNA Isolation and Real-Time qRT-PCR

Total RNA was extracted from leaf tissues using the TriPure Isolation Reagent (Roche Life Science, St. Louis, MO, USA). Total RNA (2 µg) was reverse transcribed to cDNA using Superscript II RT (Invitrogen, Waltham, MA, USA). Real-time qRT-PCR analysis was performed using one-step real-time qRT PCR (Applied Biosystems, Waltham, MA, USA) with SYBR Green, as described previously [15]. Primers for TFs, GUS, and marker genes are listed in Supplementary Table S12. The expression levels of target transcripts were normalized to the geometric mean of the housekeeping gene, *Actin*, to control the variability and further analyzed using the 2^−^^ΔΔ^*^C^*^T^ method [63]. To confirm reproducibility, three biological replicates of each assay were used for real-time qRT-PCR analysis, and three technical replicates were analyzed for each biological replicate.

### 4.12. Hormonal Treatment

For hormonal treatment, 28-day-old transgenic *N. benthamiana* plants were sprayed individually with SA (1 mM), MeJA (100 µM), and ABA (100 µM) and infiltrated with CymMV and ORSV infectious clones independently. Another set of plants was infiltrated and sprayed with a combination of viruses and hormones. 

### 4.13. Phytohormone Extraction

For phytohormone extraction from *N. benthamiana* leaves, a previously published method [64] with slight modifications was employed. Briefly, 1 g of leaves was ground into powder in liquid nitrogen. The powder was soaked in a 5 mL extraction solvent (a mixture containing 2-propanol, H_2_O, and 12 N HCl in a ratio of 2:1:0.002 by volume) and vigorously shaken for 30 min at 4 °C. Leaf tissues were then pelleted by centrifugation at 10,000× *g*, 4 °C for 10 min and further filtered using Miracloth (125 μm pore size). The resulting solution was passed through a C18 column (Avantor™ BAKERBOND™ spe Octadecyl (C18) Disposable Extraction Columns, Thermo Fisher Scientific Inc, Waltham, MA, USA) to remove the chlorophyll. Then 10 mL dichloromethane was added to the filtrate and vigorously shaken for 30 min at 4 °C, followed by centrifugation at 10,000× *g*, 4 °C for 10 min. Two phases were subsequently formed, and the lower phase containing the phytohormones mixture was collected. The solvent of the mixture was evaporated using nitrogen blow-down. The sample was re-dissolved in 100 µL methanol and stored at −80 °C until UHPLC-ESI-MS/MS analysis.

### 4.14. UHPLC-ESI-MS/MS Analysis of Salicylic Acid and Jasmonic Acid

Phytohormones were measured using a Thermo Scientific Dionex UltiMate 3000 system (Thermo Fisher Scientific Inc.) linked to an amaZon speed-ion trap mass spectrometer (Bruker, Billerica, MA, USA) equipped with electrospray ionization (ESI). Salicylic acid and jasmonic acid were separated using an ODS column (AQUITY UPLC BEH shield RP18, 1.7 μm, 2.1 × 100 mm, Waters, BA, UK) with a biphasic solvent system consisting of 0.1% (v/v) formic acid in ddH_2_O (A) and 100% acetonitrile (B) at a 0.3 mL min^−1^ flow rate. The linear gradient was set according to the following profile:0 min, 100% A; 2 min, 60% A + 40% B; 5 min, 40% A + 60% B; 13 min, 100% B, and then kept for 2 min and equilibrated for 5 min before the next injection. The injection volume used was 20 µL. The column oven temperature was set to 40 °C. The mass spectrometer parameters were as follows: 4.5 kV capillary; 500 V endplate offset voltage; 40.0 psi nebulizer pressure; 8.0 L min^−^^1^ dry gas, 230 °C dry temperature. The full scan was set at 40–300 m/z. In negative mode, ESI-MS/MS was operated in multiple reaction monitoring (MRM). MRM was set at 137–93 m/z to detect salicylic acid and 209–165 m/z to detect jasmonic acid.

### 4.15. Statistical Analysis

All the GUS quantitative experiments were performed three times. Data are presented as the mean ± SD. Treatment means were further compared by one-way analysis of variance with Student’s *t* test using GraphPad Prism 8.1.2 (GraphPad Software, La Jolla, CA, USA); differences with *p* values < 0.05 were considered significant.

## Data Availability

Not applicable.

## Supplementary Materials

The following supporting information can be downloaded at: https://www.mdpi.com/article/10.3390/ijms23179825/s1.
