# Supplementary material for: Characterization of Virus-Inducible Orchid Argonaute 5b Promoter and Its Functional Characterization in Nicotiana benthamiana during Virus Infection
Source: Int J Mol Sci. 2022 Aug 29;23(17):9825. doi: 10.3390/ijms23179825 (PMC9456093; doi:10.3390/ijms23179825)
Supplement: Supplementary file 1 [file ijms-23-09825-s001.zip › Supplementary Figures.pdf]

Supplementary Figures

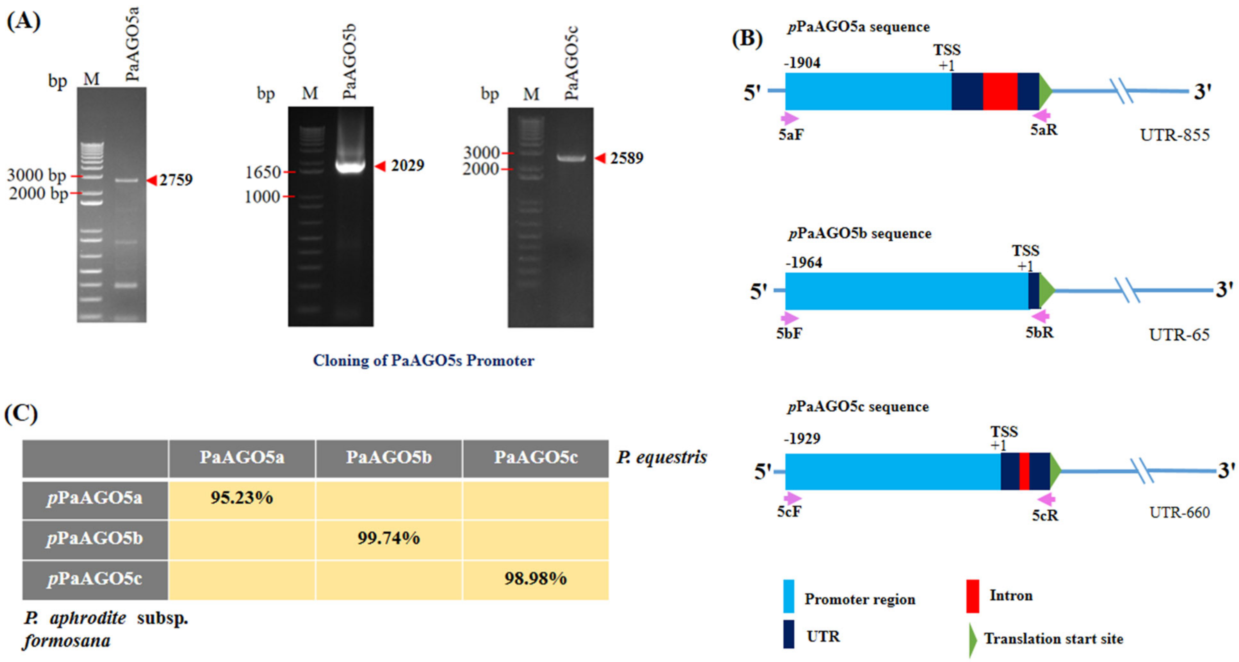

Figure S1. **(A)** PCR amplification of the promoter sequences of *p*PaAGO5a, *p*PaAGO5b, and *p*PaAGO5c. **(B)** Schematic diagram of the promoter sequences of *p*PaAGO5a, *p*PaAGO5b, and *p*PaAGO5c. **(C)** BLAST search analysis of the promoter sequences of *p*PaAGO5a, *p*PaAGO5b, and *p*PaAGO5c with promoter sequences of *P. equestris* PaAGO5a, PaAGO5b, and PaAGO5c. M: Marker; bp: Base pair; UTR: Untranslated region; TSS: Transcription start site; *p*-indicates promoter.

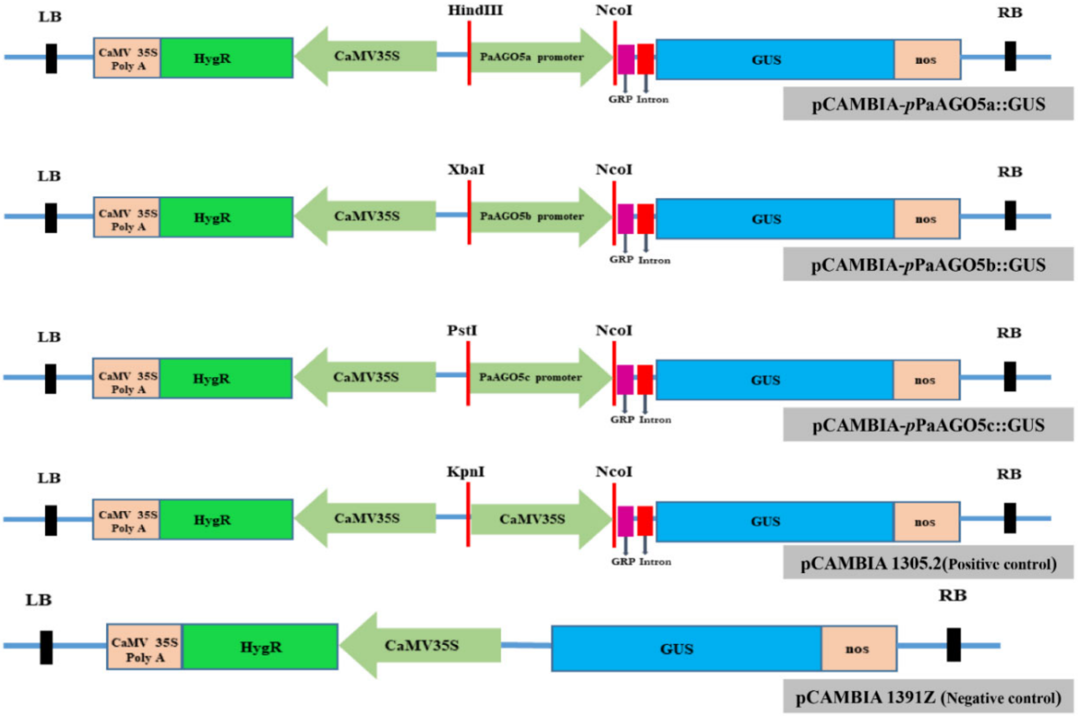

Figure S2. Schematic representation of different promoter constructs. The vector pCAMBIA1305.2 (positive control) containing right border (RB) T-DNA repeat; reporter gene GUS under the control of CaMV 35S promoter with NOS terminator; LacZ gene under the control of LacZ promoter for cloning the gene of interest; *Hyg*R gene as a selection marker under the control of CaMV 35S promoter and CaMV 35S poly A terminator; left border (LB) T-DNA repeat. (CaMV 35S: *Cauliflower mosaic virus* 35S promoter). The vector pCAMBIA1391Z does not have a CaMV 35S promoter to express the GUS reporter gene and was used as a negative control. The vector constructs pCAMBIA-pPaAGO5a::GUS, pCAMBIA-*p*PaAGO5b::GUS and pCAMBIA-*p*PaAGO5c::GUS with *p*PaAGO5a, *p*PaAGO5b and pPaAGO5c to control the expression of GUS reporter gene.

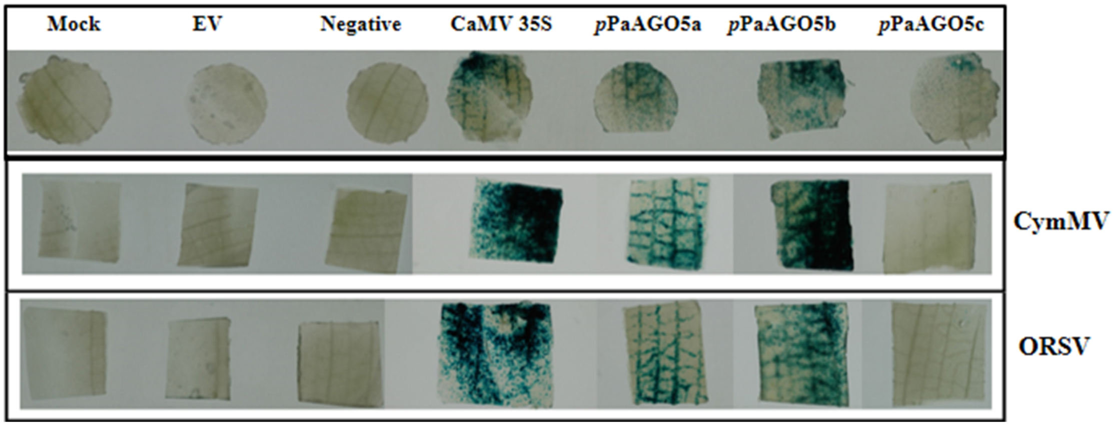

Figure S3. GUS histochemical staining of transformed *P. aphrodite* subsp. *formosana* leaves with *p*PaAGO5a, *p*PaAGO5b, *p*PaAGO5c, mock, empty vector (EV), negative and CaMV 35S under CymMV and ORSV infection at 3dpi.

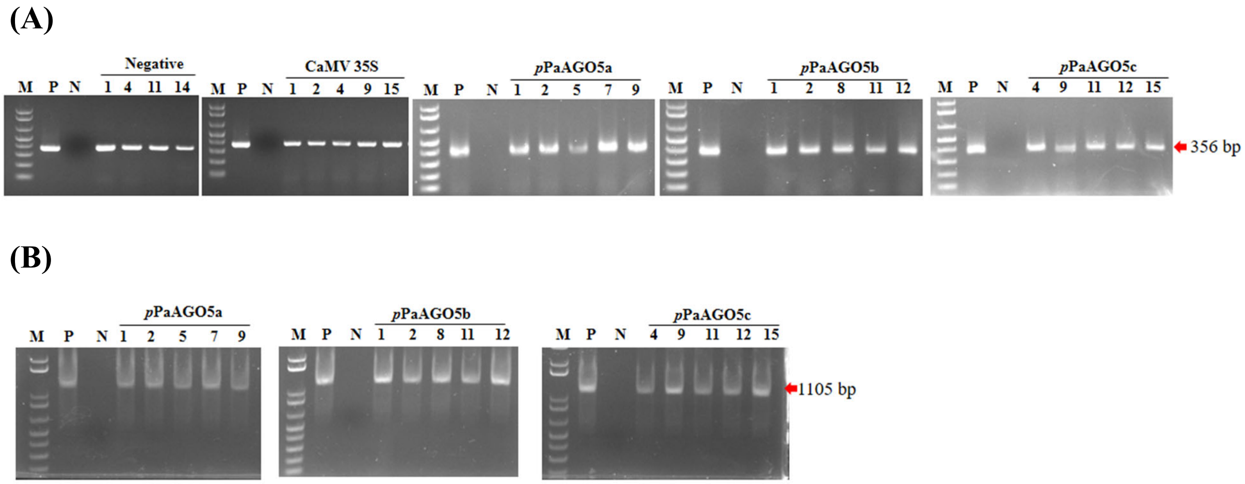

Figure S4. Confirmation of homozygous T_3 transgenic *N. benthamiana* by **(A)** *HygR* and **(B)** *GUS* gene PCR analysis. The expected length of the amplified *HygR* gene is 356 bp and *GUS* gene is 1105 bp. M: marker; P: positive control; N: negative control (distilled water used as template); Number 1-15 indicates transgenic lines for different constructs.

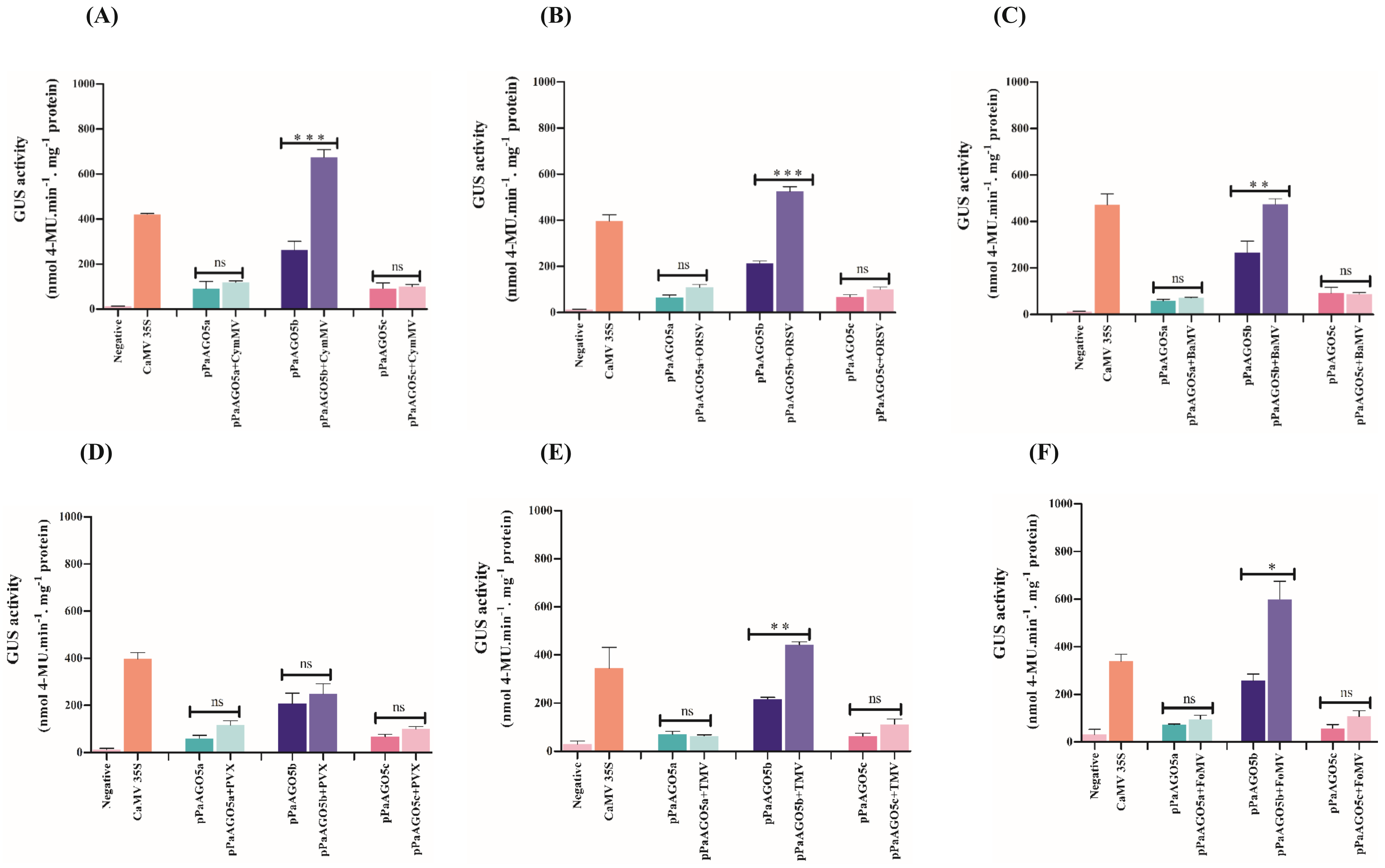

(A)
(B)
(C)
(D)
(E)
(F)
GUS activity
(nmol 4-MU.min-1. mg-1 protein)
1000
800
600
400
200
0
Negative
CaMV 35S
pPaAGO5a
pPaAGO5a+CymMV
pPaAGO5b
pPaAGO5b+CymMV
pPaAGO5c
pPaAGO5c+CymMV
pPaAGO5a+ORSV
pPaAGO5b+ORSV
pPaAGO5c+ORSV
pPaAGO5a+BaMV
pPaAGO5b+BaMV
pPaAGO5c+BaMV
pPaAGO5a+PVX
pPaAGO5b+PVX
pPaAGO5c+PVX
pPaAGO5a+TMV
pPaAGO5b+TMV
pPaAGO5c+TMV
pPaAGO5a+FoMV
pPaAGO5b+FoMV
pPaAGO5c+FoMV
ns
* * *
* *
*

Figure S5. GUS fluorescent quantitative analysis of the *p*PaAGO5a, *p*PaAGO5b and *p*PaAGO5c under **(A)** CymMV, **(B)** ORSV, **(C)** BaMV, **(D)** PVX, **(E)** TMV and **(F)** FoMV infection in transgenic *N. benthamiana* leaves at 3 dpi. Each promoter GUS construct was assayed at least three times in four independent experiments. The GUS activity, 4-MU nmol-produced min^{-1} mg^{-1} protein, is represented as the mean ± SD of each negative and CaMV 35S, *p*PaAGO5a, *p*PaAGO5b and *p*PaAGO5c. Data are mean±SD, *P<0.05, **P<0.01, ***P<0.001 by Student's *t* test. ns = not significant

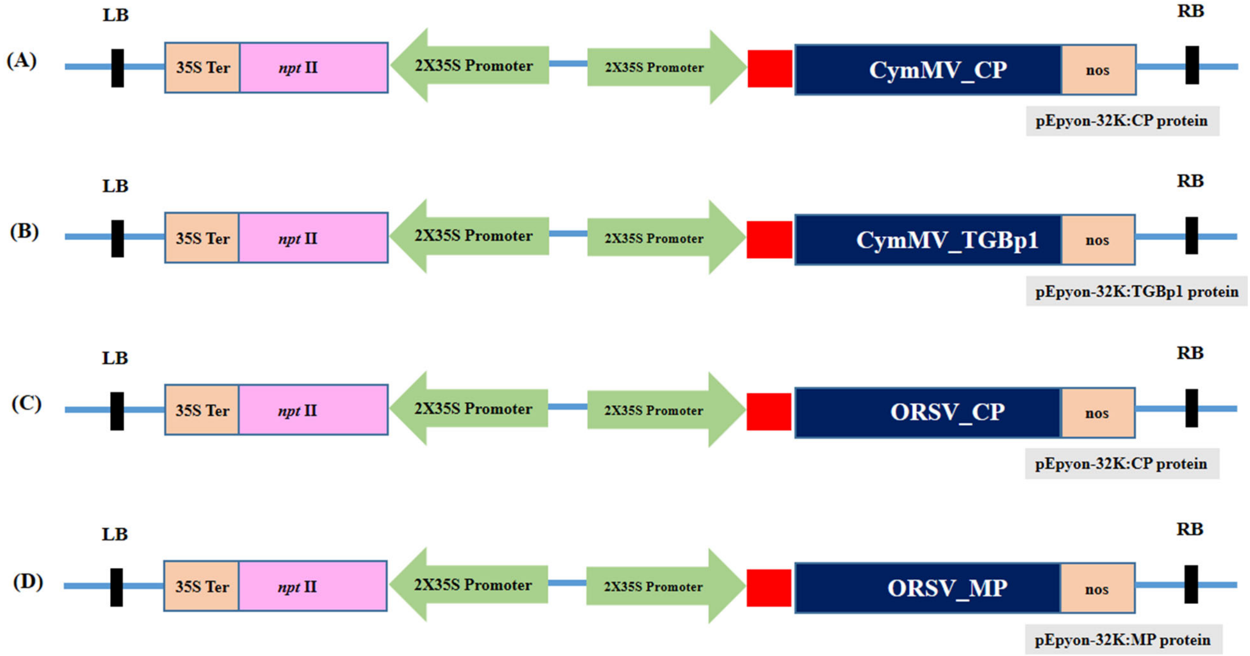

Figure S6. Schematic representation of viral genes. **(A)** CymMV_CP, **(B)** CymMV_TGBp1, **(C)** ORSV_CP and **(D)** ORSV_MP constructs. The expression vector pEPYON-32K [59] containing the right border (RB) under the control of the CaMV 35S promoter. Left border (LB) T-DNA repeat is under the control of the CaMV 35S promoter with the 35S terminator.

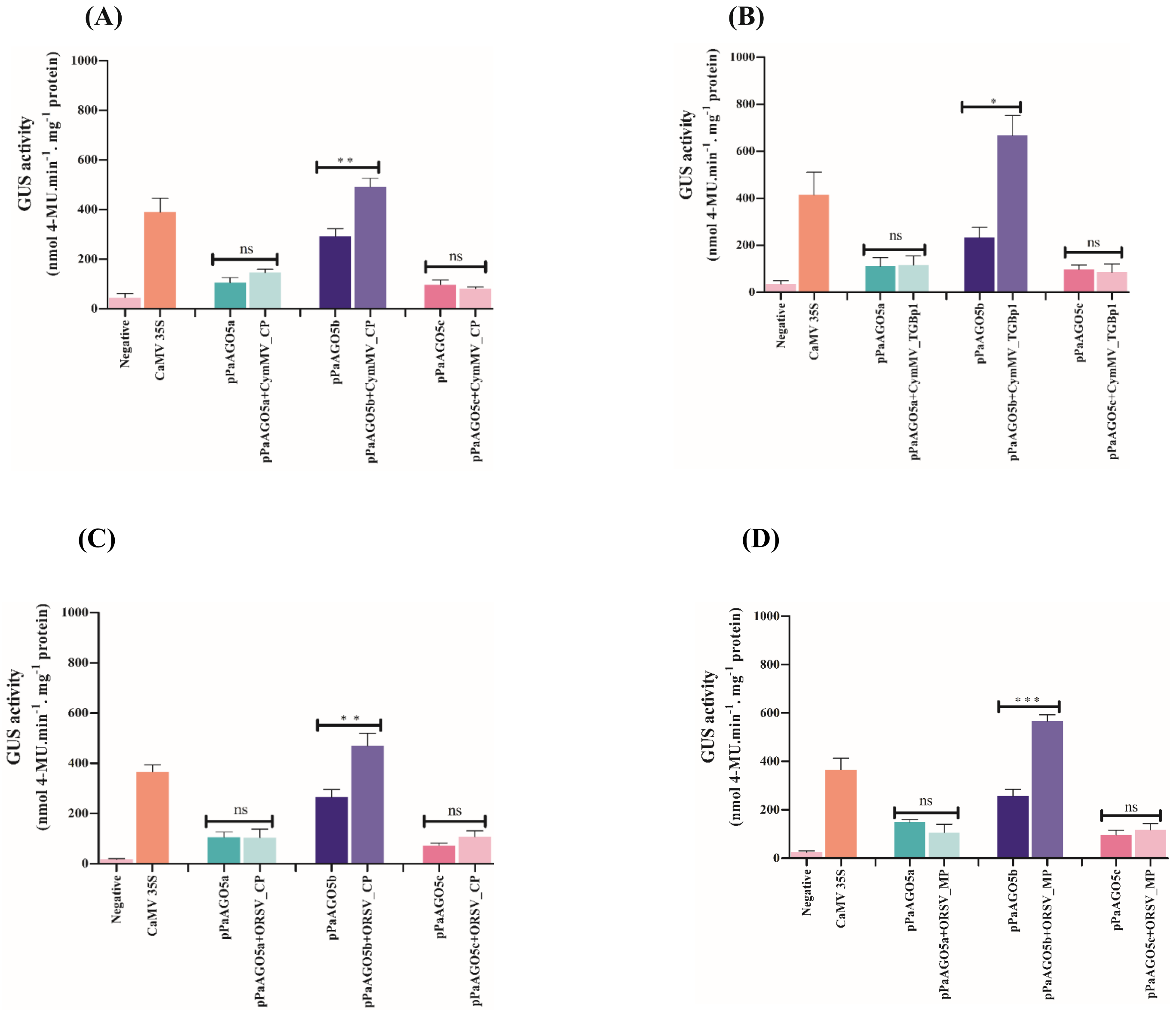

Figure S7. GUS fluorescent quantitative analysis of the *p*PaAGO5a, *p*PaAGO5b and *p*PaAGO5c under **(A)** CymMV_CP, **(B)** CymMV_TGBp1, **(C)** ORSV_CP and **(D)** ORSV_MP overexpression in transgenic *N. benthamiana* leaves at 3 dpi. Each promoter GUS construct was assayed at least three times in four independent experiments. The GUS activity, 4-MU nmol-produced min^{-1} mg^{-1} protein, is represented as the mean ± SD of each negative and CaMV 35S, *p*PaAGO5a, *p*PaAGO5b and *p*PaAGO5c. Data are mean±SD, *P<0.05, **P<0.01, ***P<0.001 by Student *t* test. ns = not significant.

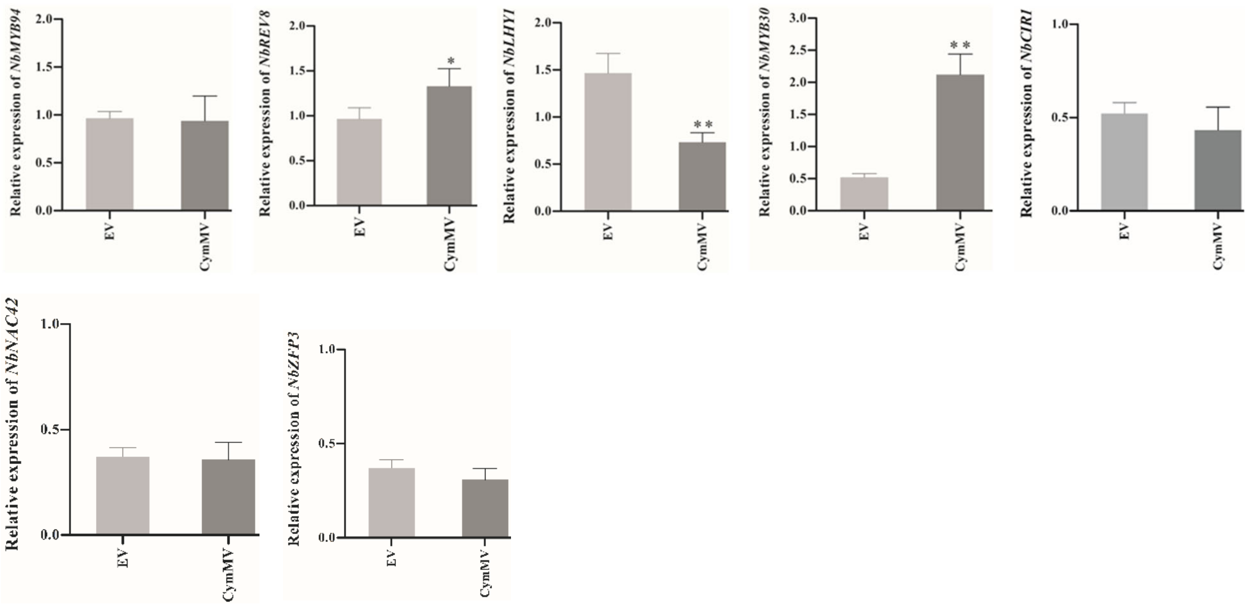

Figure S8. The accumulation of transcription factor transcripts in CymMV infected *N. benthamiana* leaves. CymMV infectious clone delivered into transgenic *N. benthamiana* leaves through agroinfiltration, respectively. The leaves were collected at 72 hours post infiltration (hpi) for total RNA extraction. The transcript accumulation of each transcription factor was examined by real-time qRT-PCR. The data are the averages from three replicates. The error bars indicate standard deviations (SD). Asterisks indicate statistical significance between independent groups assessed by Student's *t* test (*, $P < 0.05$; **, $P<0.01$). EV, empty vector (pKn); CymMV, the leaves agroinfiltrated with CymMV infectious clone.

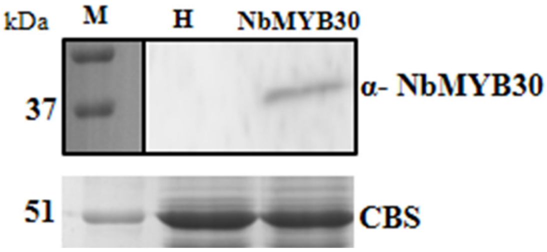

Figure S9. Western blot analysis using anti-Flag antibody confirmed the expression of pEPFlag-NbMYB30. For Western blot, the NbMYB30 was detected by respective specific anti-serum, α-NbMYB30. Rubisco were stained with Coomassie-brilliant-blue and shown as a loading control.

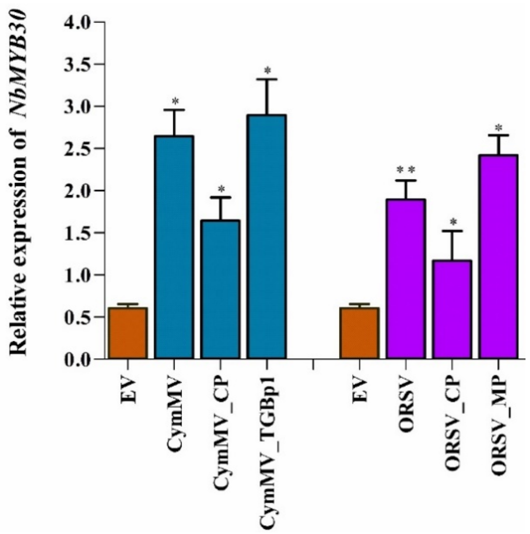

Figure S10. Transcript expression of *NbMYB30* in response to challenges of CymMV, CymMV_CP, CymMV_TGBp1, ORSV, ORSV_CP, ORSV_MP in *N. benthamiana*. The expression level of *NbMYB30* was analyzed by real time qRT-PCR in the plants infiltrated with *A. tumefaciens* harboring infectious construct of CymMV, ORSV and expression constructs of viral genes. The data are the averages from three replicates. The error bars indicate standard deviations (SD). Asterisks indicate statistical significance between independent groups assessed by Student's *t* test (*, $P < 0.05$; **, $P<0.01$). EV, empty vector (pKn).

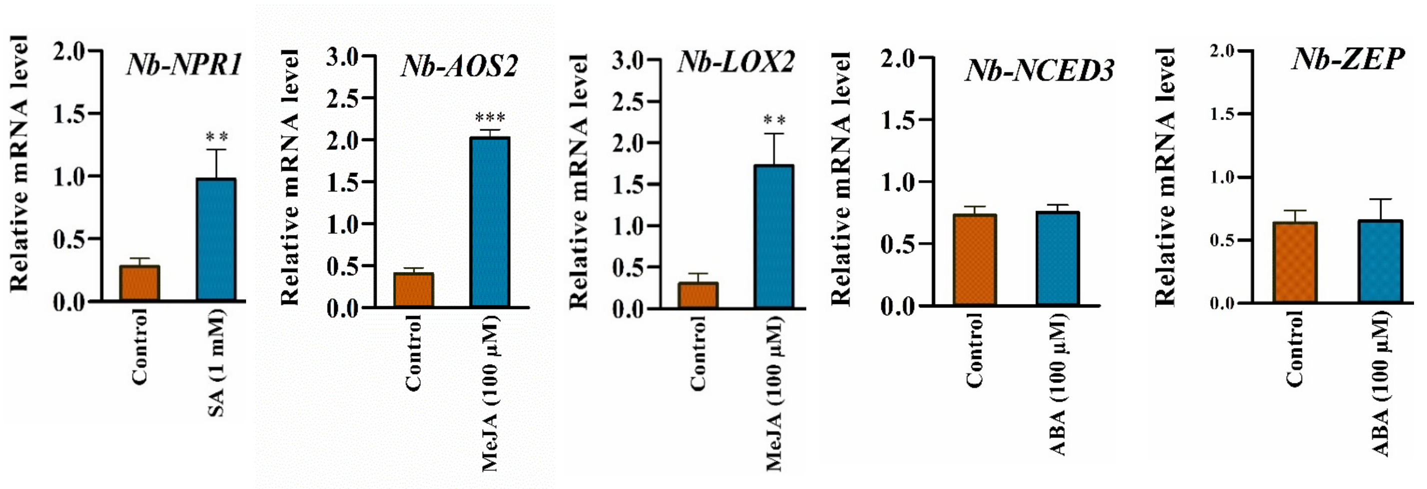

Figure S11. Effect of exogenous application of SA (1 mM), MeJA (100 µM) and ABA (100 µM). The SA marker gene *NbNPR1*, JA biosynthesis genes *NbAOS2*, *NbLOX3* were significantly increased and ABA biosynthesis genes *NbNCED3* and *NbZEP* were not enhanced in activity at 24 hpi. The data are the averages from three replicates. The error bars indicate standard deviations (SD). Asterisks indicate statistical significance between independent groups assessed by Student's *t* test (*, $P < 0.05$; **, $P<0.01$). Control, 0.001% ethanol.

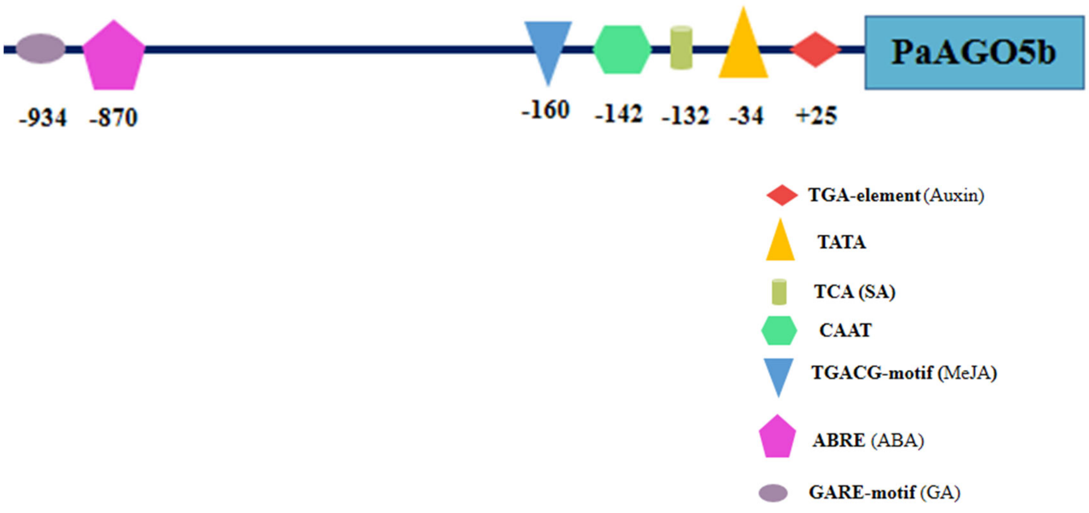

Figure S12. Hormone related *cis*-acting elements presence and their location on the *p*PaGO5b. The presence of TATA-box and CAAT-box location also mentioned.

(A)

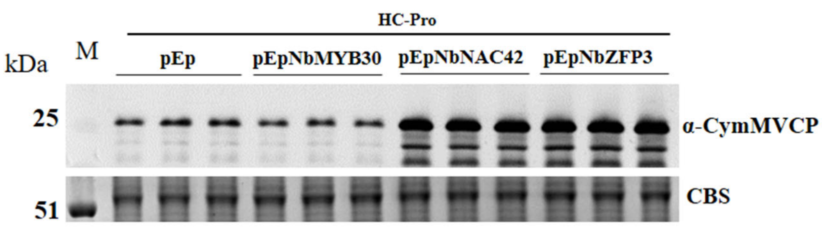

(B)

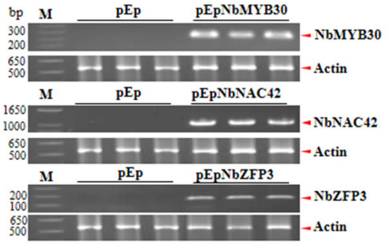

(C)

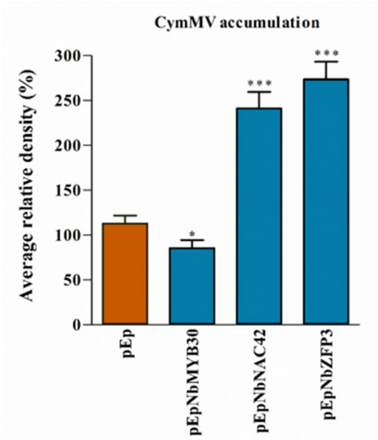

Figure S13. CymMV accumulation in the leaves expressing *N. benthamiana* transcription factors NbMYB30, NbNAC42 and NbZFP3. *P. aphrodite* subsp. *formosana* leaves were infiltrated with *agrobacterium* EHA105 harboring pEPYON 32K vector (pEp), pEpNbMYB30, pEpNbNAC42 or pEpNbZFP3 expression vectors. All the leaves were further agroinfiltrated with Hc-Pro expression vector and CymMV infectious clone. The leaves were collected at 3 dpi for further analyses. **(a)** CymMV accumulation in the leaves were analyzed by using western blot. (**b**) The intensity of each band was quantified and plotted. **(c)** The transcript expression of NbMYB30, NbNAC42 and NbZFP3 were analyzed by using real time qRT-PCR. For real time qRT-PCR, the expression levels of each transcripts, presented as normalized fold changes relative to that from EV leaves, were shown. Values are means ± SD of three biological replicates. Relative expression level; *, ** and ***, significant difference at P< 0.05, P< 0.01 and P<0.001 determined by Student's *t* test, respectively. For Western blot, the accumulation of CymMV and FLAG-tagged transcription factors were detected by specific respective anti-serum, α-CymMV CP or α-FLAG. Rubisco were stained with Coomassie-brilliant-blue and shown as a loading control.

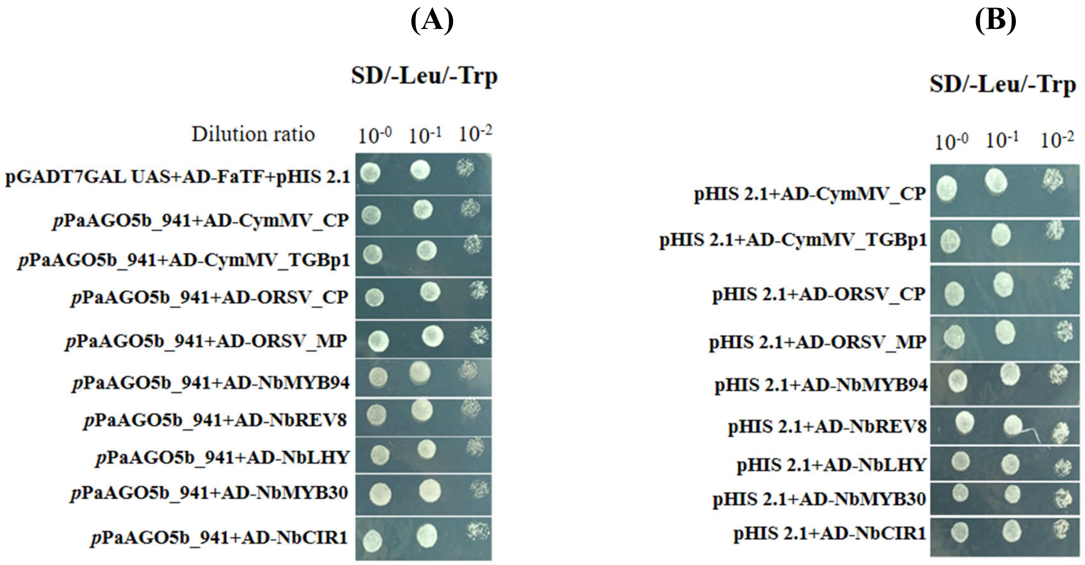

Figure S14. Physical interaction between *p*PaAGO5b_941 and viral genes or TFs in yeast cells. The full-length clones of CymMV_CP, CymMV_TGBp1, ORSV_CP, ORSV_MP, NbMYB94, NbREV8, NbLHY, NbMYB30, and NbCIR1 and were fused to pGADT74-AD and *p*PaAGO5b_941 fused to pHIS 2.1-BD were cotransformed and expressed in the yeast strain Y187. The transformed yeast cells were grown in non-selective media with histidine (SD/-Leu/-Trp) followed by incubation at 30 °C for 3 days. **(A)** The pHIS 2.1 vector and pGADT74-AD cotransformed with strawberry heat shock TF (FaTF) fused with pGBKT7 vector into yeast cells was used as the positive control. **(B)** The pHIS 2.1 vector and cotransformed with CymMV_CP, CymMV_TGBp1, ORSV_CP, ORSV_MP, NbMYB94, NbREV8, NbLHY, NbMYB30, and NbCIR1 into yeast cells and grown in non-selective media with histidine (SD/-Leu/-Trp).
