# Supplementary material for: Characterization of Virus-Inducible Orchid Argonaute 5b Promoter and Its Functional Characterization in Nicotiana benthamiana during Virus Infection
Source: Int J Mol Sci. 2022 Aug 29;23(17):9825. doi: 10.3390/ijms23179825 (PMC9456093; doi:10.3390/ijms23179825)
Supplement: Supplementary file 1 [file ijms-23-09825-s001.zip › Supplementary Tables.pdf]

Supplementary Tables

Table S1. Predicted *cis*-acting elements of the PaAGO5a gene promoter in *P. aphrodite* subsp. *formosana*.

Site Name	Sequence	Strand	Position	Number	Function
3-AF1 binding site	TAGGAGAGGAA	-	-740	1	Light responsive element
ABRE	TACGGTC<br>TACGTGTC<br>ACGTG<br>TACGGTC<br>CACGTG	+<br>+<br>+<br>-<br>-	-467, +1103<br>+1313<br>-79, -479, +1311<br>+1122<br>-80, -480	9	*Cis*-acting element involved in the abscisic acid responsiveness
ABRE3a	TACGTG	+	+1313	1	*Cis*-acting element involved in the abscisic acid responsiveness
ACE	GATACGTATG	+	-309, +2108	2	*Cis*-acting element involved in light responsiveness
ARE	AAACCA	-	-126, -133, +1731	3	*Cis*-acting regulatory element essential for the anaerobic induction
AT-rich element	ATAGAAATCAA	-	+1583	1	Binding site of AT-rich DNA binding protein (ATBP-1)
BOX-4	ATTAAT	-	-651	1	Part of a conserved DNA module involved in light responsiveness
CAAT-box	CAAT<br>CAAAT<br>CAAT<br>CAAAT<br>TGCCAAC	+<br>+<br>-<br>-<br>-	-9, -100, -426, -232, -753<br>-340, -527, -643, -659, +851<br>-329, -621, -652, -797<br>-397, -272, -530, -690<br>+816	19	Common *cis*-acting element in promoter and enhancer regions
CGTCA-motif	CGTCA	+	+2337	1	*Cis*-acting regulatory element involved in MeJA-responsiveness
DRE	GCCGAC<br>GCCGAC	+<br>-	+2755<br>+1980	2	Regulates cold- and dehydration-responsiveness

G-Box	CACGTG<br>CACGTGAAA	-<br>-	-80, -480<br>-83	3	*Cis*-acting regulatory element involved in light responsiveness
GATA-motif	GATAGGA<br>GATAGGA	+<br>-	-282<br>+1782	2	Part of a light responsive element
GT1--motif	GGTTAAT	-	-2090	1	Light responsive element
LTR	CCGAAA	-	-784, -228	2	*Cis*-acting element involved in low-temperature responsiveness
MRE	AACCTAA	-	-600	1	MYB binding site involved in light responsiveness
MYB	CAACCA<br>CAACCA	+<br>-	+2145, +2293, +2483<br>+860, +2731	5	MYB binding site involved in drought -inducibility
MYC	CATGTG<br>CATTTG	+<br>-	+877<br>-176, +1362	3	MYB binding site involved in chilling-inducibility
STRE	AGGGG<br>AGGGG	+<br>-	+2216, +2404<br>-549, +2472	4	Stress response element
TATA-box	ATTATA<br>TATAA<br>TATA<br>TACAAAA<br>TATTTAAA<br>TATTTAAA	+<br>-<br>+<br>+<br>-<br>+	-422<br>-415, -467, -620, -795<br>-422, -27, -291, -322<br>-560<br>-710<br>-150	12	Core promoter element around -30 of transcription start
TCT-motif	TCTTAC	+	-228	1	Part of a light responsive element
TGA-element	AACGAC	+<br>-	+1600<br>-757	2	Auxin-responsive element
TGACG-motif	TGACG	-	+2337	1	*Cis*-acting regulatory element involved in MeJA responsiveness
W-box	TTGACC	-	+2637	1	
WRE3	CCACCT	+	-502, +1076	2	
WUN-motif	AAATTACT	+	+2523	1	
as-1	TGACG	-	+2337	1	

Table S2. Predicted *cis*-acting elements of the PaAGO5b gene promoter in *P. aphrodite* subsp. *formosana*.

Site Name	Sequence	Strand	Position	Number	Function
AAGAA-motif	GAAAGAA	-	+638	1	Unknown function
ABRE	ACGTG<br>AACCCGG	-<br>+	+939, +1062<br>+1276	3	*Cis*-acting element involved in abscisic acid responsiveness
ABRE3a	TACGTG	-	+939	1	*Cis*-acting element involved in abscisic acid responsiveness
ARE	AAACCA<br>AAACCA	-<br>+	+309<br>+1144	2	*Cis*-acting regulatory element essential for anaerobic induction
BOX-4	ATTAAT	-<br>+	+244, +401<br>+1524	3	Part of a conserved DNA module involved in light-responsiveness
CAAT-box	CAAT<br><br>CAAT<br><br>CAAAT<br><br>CAAAT	+<br><br>-<br><br>+<br><br>-	+255, +342, +503, +613, +737,+741, +798, +948, +1248, +1252, +1386, +1651, +1750, +1917<br><br>+142, +304, +679, +699, +1403, 1515,+1764, +1786, +1817, +1959<br><br>+292, +483, +515, +762, +771, +1705, +1738, +1766<br><br>+1735, +1744	34	Common *cis*-acting element in promoter and enhancer regions
CARE	CAACTCAC	+	+1763	1	Unknown function
CGTCA-motif	CGTCA	-	+229	1	*Cis*-acting regulatory element involved in MeJA-responsiveness
G-Box	CACGTT	+	+1062	1	*Cis*-acting regulatory element involved in light responsiveness
G-box	CACGAC<br>TACGTG	+<br>-	+1123<br>+939	2	*Cis*-acting regulatory element involved in light responsiveness
GARE-motif	TCTGTTG	+	+1005	1	Gibberellin-responsive element

GATA-motif	AAGATAAGATG	+	+1556	1	Part of a light responsive element
GC-motif	CCCCCG	+	-56	1	Enhancer-like element involved in anoxic specific inducibility
GCN4-motif	TGAGTCA<br>TGAGTCA	+<br>-	+1087, +1638<br>+1569	3	*Cis*-regulatory element involved in endosperm expression
GT1-motif	TTAACC	-	+1968	1	Light responsive element
LTR	CCGAAA<br>CCGAAA	+<br>-	+174, +331, +1226<br>+1205	4	*Cis*-acting element involved in low-temperature responsiveness
MYB	CAACAG<br>CAACAG<br>TAACCA<br>CAACCA<br>CAACCA	+<br>-<br>+<br>+<br>-	+1688<br>+1004<br>+901<br>+1140, +584<br>+1625	5	MYB binding site involved in drought - inducibility
MYC	CATGTG	+	+967	1	
P-box	CCTTTTG	+	+1501	1	Gibberellin-responsive element
STRE	AGGGG	+	+1905	1	Stress response element
TATA-box	TATA<br>TATATA<br>TACAAAA<br>TTTAAATA	+<br>+<br>+<br>-	+352, +659, +746, +814, -1409, +1588<br>+657<br>+597, +669<br>-34	10	Core promoter element around -30 of transcription start
TCA	TCATCTTCAT	-	+206, +1293	2	Unknown
TCT-motif	GTAAGA	-	+1113	1	Part of a light responsive element
TGA-element	AACGAC<br>AACGAC	+<br>-	-41<br>+1787	2	Auxin-responsive element
TGACG-motif	TGACG	+	+229	1	*Cis*-acting regulatory element involved in MeJA-responsiveness
as-1	TGACG	+	+229	1	
Circadian	CAAAGATATC	+	-1384	1	*Cis*-acting regulatory element involved in circadian control

Table S3. Predicted *cis*-acting elements of the PaAGO5c gene promoter in *P. aphrodite* subsp. *formosana*.

Site Name	Sequence	Strand	Position	Number	Function
AAGAA-motif	GTAAAGAAA	+	+368	1	Unknown function
ACE	CTAACGTATT	-	-107	1	*Cis*-acting element involved in light-responsiveness
AE-box	AGAAACAA<br>AGAAACTT	-<br>-	-509,<br>-1444	2	Part of a module for light response
AT1-motif	AATTATTTTTATT	+	-950	1	Part of a light responsive module
ATC-motif	AGTAATCT	+	-1615	1	Part of a conserved DNA module involved in light-responsiveness
Box 4	ATTAAT	+	-572, -733, -961, -1362	4	Part of a conserved DNA module involved in light-responsiveness
CAAT-box	CAAT<br>CAAT<br>CAAAT<br>CAAAT	+<br>-<br>+<br>-	-48, -116, -392, -35, -150, -215, -267, -414, -568, +766, -780, +857, +872, +1349, +1360, +1476, +1493, +1723, +1739, -486, -517,-579,-168,-343, -604, +998, +1790<br>-206,-524,-202,-221, -431, -451, -473, +795, +880, +1055, +1344<br>+1270,+1369, +1777	41	Common *cis*-acting element in promoter and enhancer regions
CCAAT-box	CAACGG	+	-155	1	MYBHv1 binding site
CTAG-motif	ACTAGCAGAA	+	-547	1	Unknown function

G-box	CACGAC	-	-627	1	*Cis*-acting regulatory element involved in light-responsiveness
GARE-motif	TCTGTTG	-	-19	1	Gibberellin-responsive element
GCN4_motif	TGAGTCA	-	-874	1	*Cis*-regulatory element involved in endosperm expression
GT1-motif	TTAACC	+	-902	1	Light-responsive element
MSA-like	TCCAACGGATA	+	-112	1	*Cis*-acting element involved in cell cycle regulation
TATA-box	TATA	+	-98,-126,-147,-183, -335,-81, -88, -138, -196,-321,-381,-41, -526, -588, -664, +671, +715, +756, +809,+1096,+1425, +1531,+1562,+1573, +1698, +1752	26	Core promoter element around -30 of transcription start
TC-rich repeats	ATTCTCTAAC	-	-275, -372, 1155, -1542,	4	*Cis*-acting element involved in defense and stress responsiveness
TCT-motif	TCTTAC	+	-1155	1	Part of a light responsive element
chs-CMA1a	TTACTTAA	+	-1327	1	Part of a light responsive element

Table S4. Primers used for the cloning of *p*PaAGO5a, *p*PaAGO5b and *p*PaAGO5c.

Primer name	Primer sequence (5′–3′)
PaAGO5a_Promo-F	GCAAGCTTGGCAGCCGACGACAGCATTGG
PaAGO5a_Promo-R	GCCCATGGGTTGTGTTGGTCGAAGACTTTG
PaAGO5b_Promo-F	GCTCTAGAGTGCATTGCATATGTTTTGACA
PaAGO5b_Promo-R	GCCCATGGCTAACGAATCTTCCAGACTAC
PaAGO5c_Promo-F	GCCTGCAGGGCCTAGCTACACGAGATGAC
PaAGO5c_Promo-R	GCCCATGGATCAACTTTCCCAAACTGATG

Letters with underline (_) indicate the DNA sequence of the restriction sites.

Table S5. Primers used for the screening of transgenic *N. benthamiana* plants.

Primer name	Primer sequence (5′–3′)
GUS-F	GGCGTCTTCGACCTCAATGGC
GUS-R	GATTCATGCCATCACGCAGCG
Hyg-F	GCCATGTAGTGTATTGACCG
Hyg-R	CTTCGCCCTCCGAGAGCTG

Table S6. Primers used for the cloning of *p*PaAGO5b 5'-deletion constructs.

Primer name	Primer sequence (5′–3′)
PaAGO5b_2029-F	GCTCTAGAGTGCATTGCATATGTTTTGACA
PaAGO5b_1782-F	GCTCTAGAGTTTCATGCAAATCTTGT
PaAGO5b_1582-F	GCTCTAGATGAAATATATTATTGGAA
PaAGO5b_1182-F	GCTCTAGAATACTCCGTTTAGACTAC
PaAGO5b_941-F	GCTCTAGAAAACACGTACTTCAGAGGACAC
PaAGO5b_582-F	GCTCTAGACAAATTAGGACGCAATAC
PaAGO5b_349-F	GCTCTAGACATCGACTAGGTGTAGGG
PaAGO5b_235-F	GCTCTAGAGTGACGAGAGCGCCATATG
PaAGO5b_109-F	GCTCTAGACCCTTACCACTCAGACAT
PaAGO5b_88-F	GCTCTAGATCCGCCTTCCCCCCGTAG
PaAGO5b_65-F	GCTCTAGATGAAACGACGCTTCGCTG

PaAGO5b_Promo-R	GCCCATGGCTAACGAATCTTCCAGACTAC

Letters with underline indicate (_) the DNA sequence of the restriction sites.

Table S7. Primers used for the cloning of viral genes.

**Primer name**	**Primer sequence (5′–3′)**
CymMV_CP-F	GCCTGCAGATGGGAGAGCCCACTCCAAC
CymMV_CP-R	GCGAGCTCTTATTCAGTAGGGGGTGCAG
CymMV_TGBp1-F	GCTCTAGAATGGATTACAAGGACGACGATGACAAGGAGCTAGCGTACTTAGTTAGA
CymMV_TGBp1-R	GCGAGCTCTCAGGTGGTGGAACTAAACCTGG
ORSV_CP-F	GCTCTAGAATGTCTTACACTATTACAGAC
ORSV_CP-R	GCGAGCTCTTAGGAAGAGGTCCAAGTAAG
ORSV_MP-F	GCCTGCAGATGGATTACAAGGACGACGATGACAAGGCTCTAGTACTAAGGGATAGC
ORSV_MP-R	GCGAGCTCTCAATACGAATCAGATTGTGC

Letters with underline indicate (_) the DNA sequence of the restriction sites. Green color indicates sequence of FLAG tag antibody.

Table S8. List of TFs selected for yeast one hybrid analysis.

**ID** (From *N.benthamina*)	**TFs name**
Niben101Scf07438g03001.1	NbMYB94
Niben101Scf07223g01008.1	DNA binding protein (REVEILLE8/LHY-CCA1-LIKE5 (RVE8/LCL5)
Niben101Scf02026g01002.1	Late elongated hypocotyl and circadian clock associated-1-like protein 1
Niben101Scf01908g01004.1	Myb-related protein 30 (NbMYB30)
Niben101Scf09590g03004.1	Circadian 1 (CIR1) transcription factor

Table S9. Primers used for the cloning of *N.benthamina* TFs.

**Primer name**	**Primer sequence (5′–3′)**
NbMYB94_F	GCGAATTCATGGGAAGACCACCTTGCTGT
NbMYB94_R	GCGGATCCTTATTCAGGTGTAAAATTACC
NbREV8_F	GCGGATCCATGAACTCAAACTCTCCTTCC

NbREV8_R	GCCTCGAGTCAAGGCTTGGGCAAGCTTCT
NbLHY _F	GCGAATTCATGCAAGTGCCACACAACTATCC
NbLHY _R	GCGAGCTCTCAAATAGAAGCTTCTCCTTCC
NbMYB30_F	GCGAATTCATGGGAAGGCCTCCTTGTTGT
NbMYB30_R	GCGGATCCTTAAGTGAATATCATAGGAAG
NbCIR1_F	GCCCCGGGATGACTATACAGGATCAAAGC
NbCIR1_R	GCCTCGAGCTACAAGCATAACCGGGTTCGC

Letters with underline indicate (_) the DNA sequence of the restriction sites.

Table S10. Primers used for the cloning of pEPFlag-NbMYB30 for transient expression and *NbMYB30* for VIGS.

**Primer name**	**Primer sequence (5′–3′)**
NbMYB30_F_OE	GCCTGCAGATGGATTACAAGGACGACGATGACAAGGGAAG GCCACCTTGTTG
NbMYB30_R_OE	GCGAGCTCTCAAAGCCATTCCATGCCTATTC
NbMYB30_F_VIGS	GCGAATTCGTACAACTGCTGATTTG
NbMYB30_R_VIGS	GCGGATCCTATATCTACTAGTACATATGC

Letters with underline indicate (_) the DNA sequence of the restriction sites. Green color indicates sequence of FLAG tag antibody.

Table S11. Primers used for the cloning of pCFlag-PaMYB30 for transient expression and pKFV_PaMYB30 for VIGS.

**Primer name**	**Primer sequence (5′–3′)**
PaMYB30_F_OE	GCCTGCAGATGGATTACAAGGACGACGATGACAAGGGAAG ACCTCCTTGTTGTG
PaMYB30_R_OE	GCGAGCTCTCAATCCTGAAACAACTCTGAAG
PaMYB30_F_VIGS	ATGTTAACGTCGTCTTGAAGGAGGAG
PaMYB30_R_VIGS	ATGTTAACAAAGCGGATGGAAGCAG

Letters with underline indicate (_) the DNA sequence of the restriction sites. Green color indicates sequence of FLAG tag antibody.

Table S12. Primers used for the qRT-PCR of TFs.

**Primer name**	**Primer sequence (5′–3′)**
NbMYB94_F_qRT-PCR	CTGGAATACTCATCTGAG
NbMYB94_R_qRT-PCR	CAGAGAATCAGATTTG
NbREV8_F_qRT-PCR	GGTTGTGTCCCAAGTTG
NbREV8_R_qRT-PCR	GCGTCATCTTTTCGCAT
NbLHY_F_qRT-PCR	GCTCAAAAGTACTTCTT
NbLHY_R_qRT-PCR	GGTGGAAGGAAATTCAT
NbMYB30_F_qRT-PCR	CCTATAATTCTGATGTTTC
NbMYB30_R_qRT-PCR	GTTAAGGGTACTTCCGTC
NbCIR1_F_qRT-PCR	CGCGAAAGCTGGACCGA
NbCIR1_R_qRT-PCR	GGTTCCACTCTTCTGGAC
GUS_F_qRT-PCR	CACCGAGACCCGTGGC
GUS_R_qRT-PCR	CCGAAGCGGAGCACGA
PaMYB30_F_qRT-PCR	CTCATGGCTATATCCCCTG
PaMYB30_R_qRT-PCR	CAATTCGCTCTCCTTGAAC
NbNAC42_F_ qRT-PCR	GAACTGGAGGACAAC
NbNAC42_R_ qRT-PCR	GAACCATGTCAAACC
NbZFP3_F_ qRT-PCR	CAGAATCGTCACATG
NbZFP3_R_ qRT-PCR	GGCAGCGGAGAAGAC
NbNPR1_F_ qRT-PCR	CTGCAGATGTTGCTAAG
NbNPR1_R_ qRT-PCR	CTCCTTTGGTTAAAAGGG
NbLOX2_F_ qRT-PCR	ATATGTGCCAAGGGACGA
NbLOX2_R_ qRT-PCR	CATAGAGTTTAAGTACATC
NbAOS2_F_ qRT-PCR	GGTCTTCCGAAGGTTCTAG
NbAOS2_R_ qRT-PCR	GCTTCGTCGCGCGAAATG
NbNCED3_F_ qRT-PCR	CTCCACGGCCATTCTG
NbNCED3_R_ qRT-PCR	GAAGGTCACCAGAAGACG
NbZEP_F_ qRT-PCR	CCGCCAACCTTTAATTGG
NbZEP_R_ qRT-PCR	GAGATGATATCCACAGG
PaNPR1_F_ qRT-PCR	AAGGAGCAAGGCCAGCTGAT

PaNPR1_R_ qRT-PCR	TCTTCCGTCGCCCTACAGTAA
PaPR1_F_ qRT-PCR	GGATCATCGTCTTGCGATTT
PaPR1_R_ qRT-PCR	CCGCACAACTGTTACATGCAT
